# Loss of p21-activated kinase Mbt/PAK4 causes Parkinson-like phenotypes in *Drosophila*

**DOI:** 10.1242/dmm.047811

**Published:** 2021-06-14

**Authors:** Stephanie M. Pütz, Jette Kram, Elisa Rauh, Sophie Kaiser, Romy Toews, Yi Lueningschroer-Wang, Dirk Rieger, Thomas Raabe

**Affiliations:** 1 Medical Radiation and Cell Research, Biocenter, Am Hubland, University of Würzburg, D-97074 Würzburg, Germany; 2 Neurobiology and Genetics, Biocenter, Am Hubland, University of Würzburg, D-97074 Würzburg, Germany

**Keywords:** *Drosophila*, Parkinson's disease, Mbt, PAK4, Negative geotaxis, Sleep fragmentation, Life expectancy, Emotional behavior, Dopaminergic PAM cluster neurons

## Abstract

Parkinson's disease (PD) provokes bradykinesia, resting tremor, rigidity and postural instability, and also non-motor symptoms such as depression, anxiety, sleep and cognitive impairments. Similar phenotypes can be induced in *Drosophila melanogaster* through modification of PD-relevant genes or the administration of PD-inducing toxins. Recent studies correlated deregulation of human p21-activated kinase 4 (PAK4) with PD, leaving open the question of a causative relationship of mutations in this gene for manifestation of PD symptoms. To determine whether flies lacking the PAK4 homolog Mushroom bodies tiny (Mbt) show PD-like phenotypes, we tested for a variety of PD criteria. Here, we demonstrate that *mbt* mutant flies show PD-like phenotypes including age-dependent movement deficits, reduced life expectancy and fragmented sleep. They also react to a stressful situation with higher immobility, indicating an influence of Mbt on emotional behavior. Loss of Mbt function has a negative effect on the number of dopaminergic protocerebral anterior medial (PAM) neurons, most likely caused by a proliferation defect of neural progenitors. The age-dependent movement deficits are not accompanied by a corresponding further loss of PAM neurons. Previous studies highlighted the importance of a small PAM subgroup for age-dependent PD motor impairments. We show that impaired motor skills are caused by a lack of Mbt in this PAM subgroup. In addition, a broader re-expression of Mbt in PAM neurons improves life expectancy. Conversely, selective Mbt knockout in the same cells shortens lifespan. We conclude that mutations in Mbt/PAK4 can play a causative role in the development of PD phenotypes.

## Abstract (precis)

**Summary:** Loss of p21-activated kinase Mbt (the PAK4 homolog) causes Parkinson-like phenotypes in *Drosophila*, including age-dependent movement deficits, shortened life expectancy and fragmented sleep. Generation of dopaminergic neurons from neural progenitors during development is impaired.

## INTRODUCTION

Parkinson's disease (PD) is a common neurodegenerative disease and the most prevalent movement disorder. PD is mainly characterized by motor symptoms such as bradykinesia, resting tremor, rigidity and postural instability, but is also accompanied by non-motor symptoms including depression, anxiety, olfactory dysfunction, sleep disorders and cognitive impairments (Kalia and Lang, 2015; Poewe et al., 2017; Postuma et al., 2015; Vogt Weisenhorn et al., 2016). It is generally accepted that motor symptoms are caused by the loss of dopaminergic (DA) neurons in the substantia nigra pars compacta (SNpc) (Raza et al., 2019; Vogt Weisenhorn et al., 2016). The origin of non-motor symptoms has not been investigated in detail, but these symptoms frequently appear much earlier during etiopathology (Kalia and Lang, 2015; Poewe et al., 2017) and may be partially associated with dysfunction of non-DA neurons (Obeso et al., 2010). Not all symptoms appear in all patients, because PD is very heterogeneous, and the course of the disease varies depending on the subtype (Obeso et al., 2010; Poewe et al., 2017). To explain PD pathology, Braak and co-workers proposed a model in which Lewy bodies (aggregations of proteins including α-synuclein and ubiquitin) form and spread in the brain (Braak et al., 2003). However, this model only explains the symptoms of some PD variants (Obeso et al., 2010).

In addition to life restrictions caused by the symptoms, PD patients die earlier because there are no therapeutic approaches that slow down the neurodegenerative process (Fox et al., 2018; Golbe and Leyton, 2018; Kalia and Lang, 2015). The prevalent treatment of PD is still symptom based and not cause driven (Fox et al., 2018; Obeso et al., 2010). Recently, induced pluripotent stem cell approaches have been developed, which provide the basis for future dopamine cell replacement strategies (Parmar et al., 2020; Raza et al., 2019). The cause of PD remains unclear, although some genetic and environmental factors are known to increase the risk of PD. Among the genetic factors are mutations in the genes α-synuclein, leucine-rich repeat kinase 2 (*LRRK2*), parkin (*PRKN*), PTEN-induced putative kinase 1 (*PINK1*) and *DJ-1* (also known as *PARK7*) (Kalia and Lang, 2015; Poewe et al., 2017). Thus, significantly more knowledge of the cellular, molecular and genetic relationships is necessary for better differentiation of PD variants and development of effective cause-driven therapies.

A wide range of PD-like phenotypes can be induced in vertebrate and invertebrate animal models (Breger and Fuzzati Armentero, 2019; Dung and Thao, 2018; Taguchi et al., 2020). Although none of these models fully recapitulates all phenotypes of the human disease, the models enable assessment of the diverse cellular, molecular and genetic relationships of PD. In *Drosophila*, toxin-induced and genetically induced subtypes were generated, for example, through administration of rotenone, mutations in *Pink1* or *parkin*, or expression of human α-synuclein (Botella et al., 2009; Hewitt and Whitworth, 2017; Xiong and Yu, 2018). Also some considerations have been made as to how idiopathic PD could be examined (Nagoshi, 2018).

Recent studies associated the p21-activated kinases (PAKs) PAK4 and PAK6 with PD (Civiero et al., 2017; Civiero and Greggio, 2018; Danzer et al., 2007; Won et al., 2016). The six different PAK proteins expressed in vertebrate species are classified into group 1 (PAK1-3) and group 2 (PAK4-6), based on their structural properties and mode of kinase activation (Kumar et al., 2017). Whereas PAK6 activity protects against LRRK2^G2019S^-induced PD-linked phenotypes (Civiero et al., 2017), PAK4 activity is influenced by α-synuclein. In detail, PAK4 activity is an important prerequisite for the survival of DA neurons in rodents. Oligomeric α-synuclein, the major component of Lewy bodies in PD, inhibits PAK4 activity (Danzer et al., 2007; Won et al., 2016). In rat PD models, expression of activated PAK4 prevented DA neuron loss and movement disorders (Won et al., 2016). Moreover, reduced PAK4 levels and activity were observed in postmortem human PD brains (Won et al., 2016). Rat PAK4 phosphorylates CRTC1 [CREB (adenosine 3′,5′-monophosphate response element-binding protein)-regulated transcription coactivator] at serine 215 to stimulate CREB-induced transcription. PAK4–CRTC1–CREB signaling mediates the neuroprotective effects, at least in part, by upregulation of anti-apoptotic Bcl-2 expression (Won et al., 2016).

Although these studies implicate PAK4 as an important mediator in PD-associated signaling processes, the question arises whether PAK4 function in DA neurons is restricted to a neuroprotective role. Furthermore, do loss-of-function mutations in PAK4 have a causative role in the development of PD symptoms? However, complete absence of PAK4 causes lethality at embryonic stage (Qu et al., 2003). In contrast, flies lacking Mushroom bodies tiny (Mbt), which represents the only group 2 PAK protein in *Drosophila melanogaster*, with closest homology to PAK4, are viable (Melzig et al., 1998; Pütz, 2019). Wild-type flies express Mbt in the brain, detected both at the mRNA level and by means of proteomics (Aradska et al., 2015; Leader et al., 2018). This offers the opportunity to study *mbt* mutant flies for progressive, age-dependent PD-like phenotypes using a variety of behavioral assays. Furthermore, cell-type-specific re-expression or knockout of *mbt* allows us to link phenotypes to specific subclasses of DA neurons in the *Drosophila* brain. Here, we focused on protocerebral anterior medial (PAM) cluster neurons (Kasture et al., 2018). Several studies emphasized the importance of PAM neurons for the climbing ability of *Drosophila* and their relationship to progressive motor impairments in PD models (Bou Dib et al., 2014; Riemensperger et al., 2013; Tas et al., 2018). We now provide evidence that Mbt function is required in these neurons to prevent age-dependent loss of this motor function without affecting cell survival during adulthood to a large degree. In addition, loss of Mbt function causes alterations at the behavioral level, including sleep fragmentation, one of the most common non-motor symptoms observed in PD patients.

## RESULTS

### Criteria to test for a PD-like phenotype in *Drosophila*

To answer the question of whether a particular fly genotype expresses a PD-like phenotype, it must first be clarified whether and how PD symptoms manifest in *Drosophila*. Therefore, we evaluated *Drosophila* PD studies and prepared a table including PD symptoms in humans, related phenotypes in *Drosophila* and the methods that are used to investigate these phenotypes (Table 1). For many PD symptoms, corresponding phenotypes can be induced in *Drosophila* PD models by genetic alterations or toxin application (Botella et al., 2009; Hewitt and Whitworth, 2017; Xiong and Yu, 2018). However, in contrast to PD patients, loss of DA neurons was not consistently observed in *Drosophila* PD models (Botella et al., 2009; Navarro et al., 2014). This checklist allows the evaluation of the sum of observed phenotypes in relation to PD, but also emphasizes the penetrance and expressivity of the various phenotypes. These variabilities should be considered for the analysis of new candidate genes.

**Table 1.**
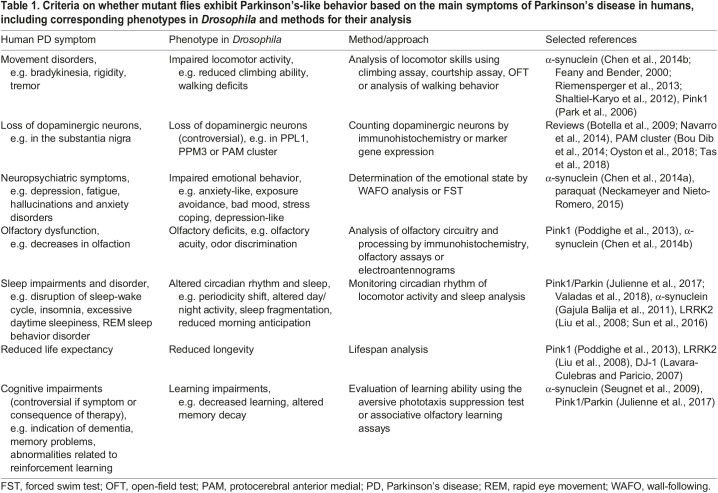
Criteria on whether mutant flies exhibit Parkinson's-like behavior based on the main symptoms of Parkinson's disease in humans, including corresponding phenotypes in *Drosophila* and methods for their analysis

To investigate whether loss of Mbt function in *Drosophila* leads to Parkinson-like disease symptoms, we therefore tested for movement deficits (climbing ability), sleep impairment, emotional behavior [wall-following (WAFO) behavior and forced swim test (FST) stress coping], loss of DA neurons and life expectancy. Because *mbt* emerged in a genetic screen for genes involved in olfactory memory formation (Walkinshaw et al., 2015) and is required for proper development of the mushroom bodies as the major integration center for associative olfactory learning (Melzig et al., 1998), cognitive impairments were not examined in our analysis.

### Characterization of Parkinson-like behaviors of *mbt* mutant flies

#### Loss of Mbt leads to reduced climbing ability

**Fig. 1.**
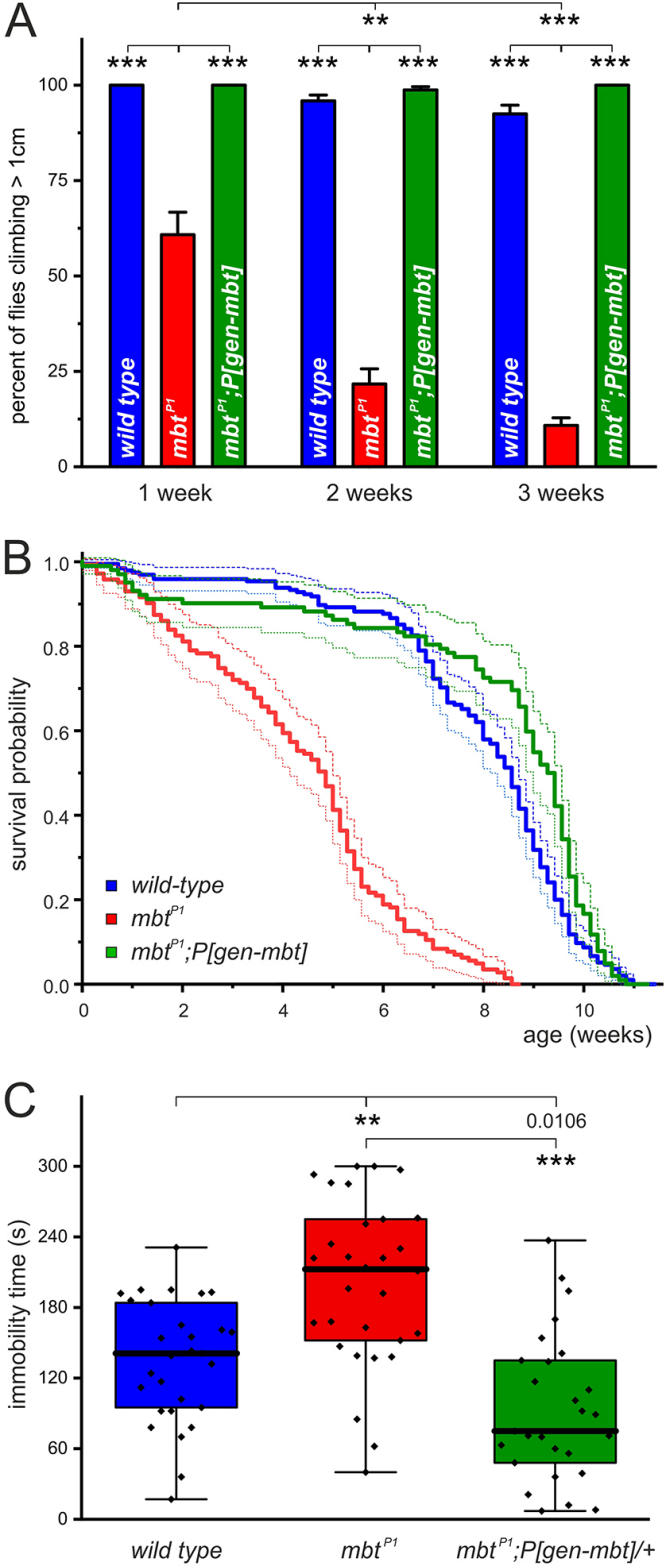
**Climbing ability, lifespan analysis and immobility values of *mbt^P1^* flies.** (A) Changes in climbing performance within the first 3 weeks of life of *mbt^P1^* flies (red) compared to wild-type (blue) and *mbt^P1^;P[gen-mbt]* (green) animals. Depicted are mean±s.e.m. of ten cohorts per genotype and age. (B) Comparison of survival probability of *mbt^P1^* (red, *n*=195), wild-type (blue, *n*=143) and *mbt^P1^;P[gen-mbt]* (green, *n*=102) flies. Statistical analysis of the survival data was performed using Kaplan–Meier analysis and the log-rank test followed by Bonferroni correction. Animals of the *mbt^P1^* genotype have a significantly reduced life expectancy compared to both control lines (*P*=0), and wild-type versus *mbt^P1^;P[gen-mbt]* flies show only small differences (*P*=0.0204). (C) Immobility time of *mbt^P1^* flies (red, *n*=30) in the forced swim test compared to wild-type (blue, *n*=29) and *mbt^P1^;P[gen-mbt]/+* (green, *n*=27) animals represented as box plots (median, quartiles, minimum/maximum values). Total analysis time was 300 s. **P<0.001, ***P<0.0001 (Mann–Whitney test followed by Bonferroni correction).

Movement disorders such as bradykinesia, resting tremor, rigidity and postural instability are characteristic symptoms that each of us associates with PD. Looking at *mbt^P1^* (null allele for *mbt*) flies, it is obvious that their movements are conspicuously different from those of wild-type flies. To quantify these differences in locomotion, the startle-induced climbing assay was performed, which is commonly used in *Drosophila* PD models (Feany and Bender, 2000; Park et al., 2006; Riemensperger et al., 2013). Upon tapping flies to the bottom of a vertically placed 16 cm experimental tube, the majority of wild-type flies reach the top within 10 s. In contrast, *mbt^P1^* flies do not stand up as quickly as controls after tapping, and even young *mbt^P1^* flies are very bad climbers, often not even reaching half the height within the same time interval. To evaluate how many flies are at least able to initiate climbing, we determined the percentage of flies that reached at least 1 cm in 10 s. To investigate a possible age-dependent decline in startle-induced climbing response, we tested flies 1, 2 or 3 weeks after eclosion. At all three time points, nearly 100% of wild-type flies reached the height of 1 cm (Fig. 1A). This result fits with previous studies showing that the capability of wild-type flies to reach the most upper zone of a vertical tube is only mildly affected even after 3 weeks of life before it strongly declines with increasing age (Feany and Bender, 2000; Vaccaro et al., 2017). In contrast to wild type, the climbing performance of *mbt^P1^* flies was already significantly impaired in 1-week-old animals (Fig. 1A). Progressive decline in climbing ability of *mbt^P1^* flies was observed after 2 and 3 weeks, with 25% reaching 1 cm at day 14 and a further reduction to 10% at day 21. To verify that progressive impairment in climbing ability is caused by loss of Mbt function, we tested *mbt^P1^* flies carrying, in addition, a genomic *mbt* transgene, *P[gen-mbt].* These flies performed like wild-type flies at all three time points (Fig. 1A).

**Fig. 2.**
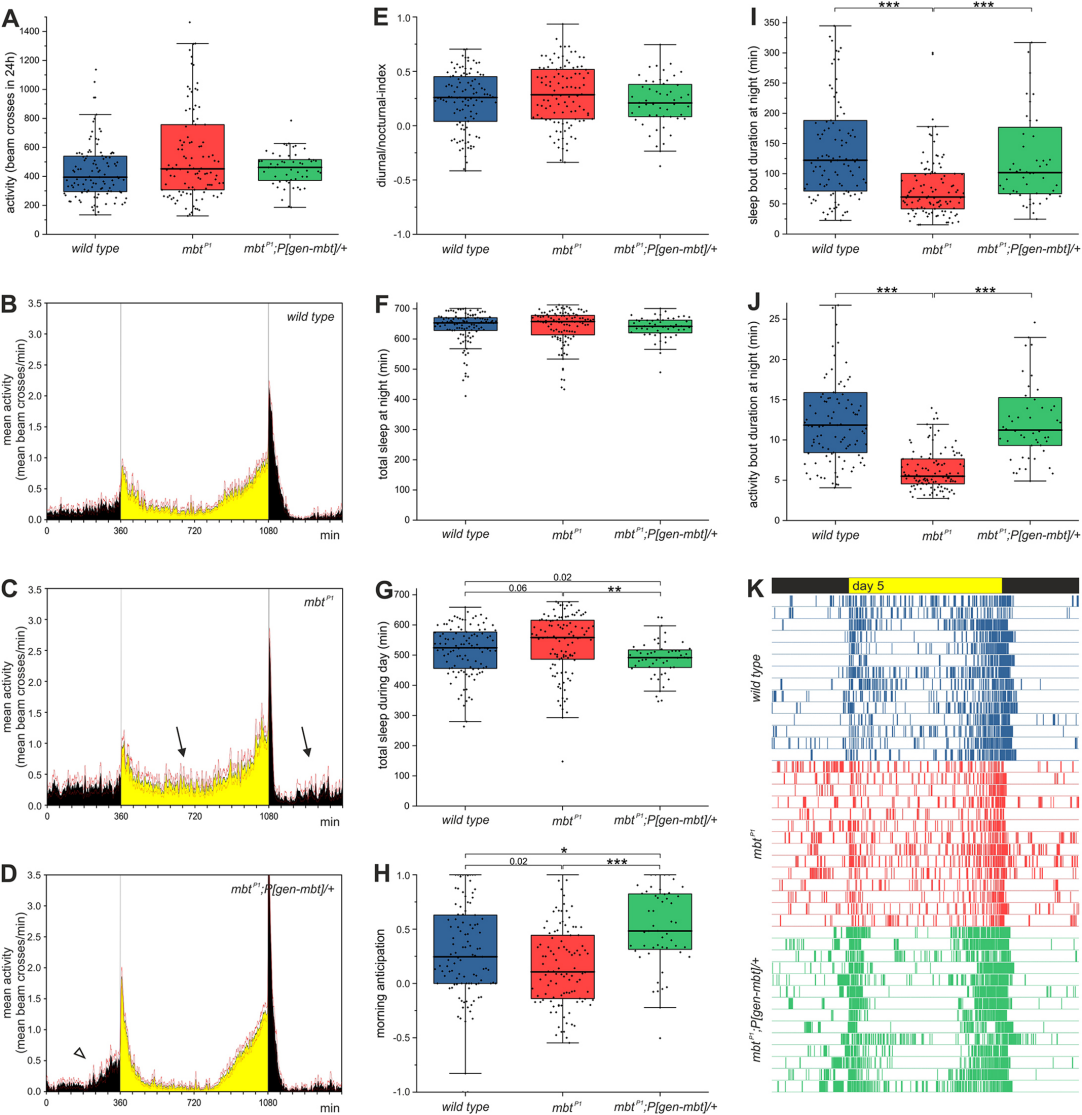
**Daily activity profiles and sleep patterns of *mbt^P1^* flies.** (A) Activity within 24 h calculated as the mean number of light beam crosses during several days of recording. (B-D) Mean activity profiles of *mbt^P1^* flies (C) compared to wild-type (B) and *mbt^P1^;P[gen-mbt]/+* (D) animals. The arrows indicate time intervals with increased activity in *mbt^P1^* flies compared to controls, and the arrowhead points to the distinct morning anticipation of *mbt^P1^;P[gen-mbt]/+* flies. (E-K) Selected activity and sleep parameters depicted as box plots: diurnal/nocturnal index (E); total sleep at night calculated as the sum of sleep minutes (F); total sleep during the day (G); morning anticipation (H); mean duration of sleep phases at night (I); mean duration of awakenings at night (J); single-day actograms (day 5) of 14 randomly selected flies per genotype (K). The colored bars represent active minutes. Sample sizes for A-J are *n*=107 wild-type (blue), *n*=106 *mbt^P1^* (red) and *n*=52 *mbt^P1^;P[gen-mbt]/+* (green) flies. The box plots indicate the median and quartile values; the whiskers are drawn up to a maximum of 1.5 times the interquartile range or the respective minimum/maximum values. Outliers are indicated. *P<0.01, **P<0.001, ***P<0.0001 (Mann–Whitney test followed by Bonferroni correction).

In this context, the question arises whether spontaneous locomotion is affected in *mbt* mutants. Spontaneous activity and startle-induced reactivity are distinct behavioral responses. Generating spontaneous activity is considered as a critical element to receive more relevant sensory input and to accomplish adaptive behavioral choices (Brembs, 2009; Heisenberg, 2015). Recordings of spontaneous activity throughout 3 days using a *Drosophila* Activity Monitor (DAM) system displayed no difference between wild-type, *mbt^P1^* and *mbt^P1^;P[gen-mbt]* flies (Fig. 2A). Regardless of genotype, spontaneous locomotion gradually declines during lifetime, with a short period of hyperactivity a few hours before death (S.M.P. and D.R., unpublished observation).

In summary, we can distinguish differential effects of Mbt on locomotion. Spontaneous activity is independent of Mbt function whereas locomotor reactivity shows two Mbt-dependent phenotypes: an early component with generally reduced climbing ability and an age-dependent, progressive component.

#### Loss of Mbt leads to a shortened lifespan

PD patients have a reduced life expectancy, regardless of medical treatment or not (Golbe and Leyton, 2018). Likewise, reduced lifespan can be observed in several fly models of PD, including DJ-1 (also known as DJ-1α), Pink1 and Parkin (Basil et al., 2017; Lavara-Culebras and Paricio, 2007; Liu et al., 2008; Poddighe et al., 2013). To address the question whether loss of Mbt influences lifespan, the survival rate of *mbt^P1^* flies was determined and compared to that of wild-type as well as *mbt^P1^;P[gen-mbt]* animals. About 80% of the wild-type flies survived until day 50; afterwards, the death rate increased. In contrast, *mbt^P1^* flies started to die with a rather constant, high rate from the time point of eclosion (Fig. 1B). The median life expectancy for *mbt^P1^* flies was 34 days, whereas that of wild-type flies was ∼60 days (Fig. 1B). Furthermore, the maximum life expectancy of *mbt^P1^* mutant flies (61 days) was reduced compared to that of wild-type flies (80 days). Flies carrying the *P[gen-mbt]* rescue construct in the *mbt^P1^* background had similar median (66 days) and maximum (79 days) life expectancies to those of wild-type flies. Therefore, Mbt is necessary for normal longevity in *Drosophila*.

#### Loss of Mbt does not affect WAFO behavior in the open-field test (OFT) but correlates with higher immobility in the FST

A further observation in PD patients is the frequent occurrence of neuropsychiatric symptoms, including depression, fatigue, hallucinations and anxiety (Jankovic, 2008; Postuma et al., 2015). All are typical preclinical characteristics that often appear before PD diagnosis (Chaudhuri et al., 2006). Anxiety, including panic attacks and social phobias, affects up to 60% of patients (Schapira et al., 2017). In rodents, the OFT is one standard test to assess emotional reactivity and explorative behavior (Prut and Belzung, 2003). Flies also express basic emotions such as fear and anger (Anderson and Adolphs, 2014; Gu et al., 2019) and show a strong WAFO behavior in the OFT (Soibam et al., 2012). The avoidance of the center part of the arena by flies is interpreted either as a preference to explore boundaries to find escape routes or as anxiety-like behavior (centrophobism) (Chen et al., 2014a; Mohammad et al., 2016; Soibam et al., 2012). Aged flies expressing human α-synuclein showed walking deficits and increased WAFO, suggesting elevated anxiety-like behavior like in PD patients (Chen et al., 2014a).

Behavioral screens uncovered common molecular players in anxiety pathways in mice and flies (Mohammad et al., 2016), including *twinstar/Cfl1*, a potential downstream target of Mbt signaling (Menzel et al., 2007). Further studies with PAK proteins in mice correlated PAK1, but not PAK5 and PAK6, with anxiety-related phenotypes (Furnari et al., 2013; Hayashi et al., 2007). To clarify this issue for Mbt, we tracked the movements of single wild-type, *mbt^P1^* and *mbt^P1^;P[gen-mbt]/+* flies in circular 1 cm diameter chambers and calculated the median distance to the arena wall. Although we did not observe significant differences between genotypes in WAFO behavior (Fig. S1), we again noticed movement coordination deficits in *mbt^P1^* flies. These observations do not support a link between Mbt and anxiety as one of the basic emotional responses affected in PD patients, but again highlighted the impact of Mbt on the motor skills of flies.

In addition to anxiety, depression is one of the most common neuropsychiatric symptoms of PD (Chaudhuri et al., 2006; Goodarzi et al., 2016). To investigate depression-like behavior in the fly, the FST was used in a previous study (Neckameyer and Nieto-Romero, 2015). Similar to rodents, higher immobility values in this assay were interpreted either as lesser capability in stress coping, negative mood or a more depressive-like behavior (Commons et al., 2017; Molendijk and de Kloet, 2019; Petit-Demouliere et al., 2005). Regarding PD, further evidence for the FST as a suitable assay came from the observation that exposure to paraquat (a PD-inducing toxin) increases immobility values both in flies and in mice (Neckameyer and Nieto-Romero, 2015; Rudyk et al., 2019). To address the question how *mbt^P1^* flies react in a stress situation, we measured the time flies were immobile during FST. *mbt^P1^* animals showed higher immobility values in FST compared to wild-type and *mbt^P1^;P[gen-mbt]/+* flies (Fig. 1C). From this, our conclusion is that *mbt^P1^* animals, depending on interpretation, have a decreased ability to cope with stress, a negative mood or are more depressive like.

#### Loss of Mbt affects sleep patterns

The most common non-motor symptoms of PD are sleep problems, affecting at least two-thirds of patients (Chaudhuri et al., 2006; Jankovic, 2008; Kalia and Lang, 2015). Sleep impairments and disturbances include insomnia, daytime sleepiness and disruption of the sleep-wake cycle, in particular sleep fragmentation and rapid eye movement (REM) sleep behavior disorder (RBD). Sleep interruptions like RBD frequently arise many years before PD becomes diagnosed and are an early indication (Högl et al., 2018; Jankovic, 2008). Sleep mechanisms are well conserved (Allada et al., 2017; Beckwith and French, 2019; Dubowy and Sehgal, 2017; Helfrich-Förster, 2018), and changes in diurnal activity and sleep patterns also occur in *Drosophila* models for PD (Jahromi et al., 2015; Julienne et al., 2017; Liu et al., 2008; Sun et al., 2016; Valadas et al., 2018). To investigate whether the loss of *mbt* leads to alterations in daily activity and sleep patterns, locomotor activities of *mbt^P1^*, wild-type and *mbt^P1^;P[gen-mbt]/+* flies were monitored with the DAM system. Evaluating the mean activity profiles (Fig. 2B-D) uncovered increased activity of *mbt^P1^* flies between the usual morning and evening peaks, both at night and during the day (arrows in Fig. 2C), without changing the overall activity (beam crosses) within 24 h (Fig. 2A) or the diurnal/nocturnal index (Fig. 2E). These findings indicated that loss of Mbt function affects either the duration, intensity or distribution of activity phases.

Remarkably, several characteristics of sleep pattern defects (daytime sleepiness, dawn anticipation and sleep fragmentation) associated with PD were observed in *mbt^P1^* mutant flies. In detail, although no differences in overall sleep were observed between the genotypes at night (Fig. 2F), daytime sleepiness in *mbt^P1^* flies was slightly elevated (Fig. 2G). Another outstanding finding of sleep analysis in PD fly models is the diminished anticipation of light changes at dusk and dawn (Gajula Balija et al., 2011; Valadas et al., 2018). Wild-type flies increase their activity before lights on and off (Fig. 2B) (Grima et al., 2004; Valadas et al., 2018). In *mbt^P1^* flies, morning anticipation was slightly reduced (Fig. 2H), but not significantly different from that of wild type. In the case of *mbt^P1^;P[gen-mbt]/+* flies, morning anticipation was even more pronounced (Fig. 2H and arrowhead in Fig. 2D).

The most striking difference in the sleep behavior of *mbt^P1^* flies compared to that of wild-type and *mbt^P1^;P[gen-mbt]/+* flies was fragmented sleep at night. Sleep fragmentation in *Drosophila* is characterized by shortened sleep phases that alternate with brief awakenings (Valadas et al., 2018). In comparison to wild-type and *mbt^P1^;P[gen-mbt]/+* animals, sleep bout duration was considerably reduced in *mbt^P1^* flies, especially at night (Fig. 2I). The second feature of sleep fragmentation also applied to *mbt^P1^* flies: awakenings at night were very short, lasting only a few minutes (Fig. 2J). Single-day actograms at day 5 of 14 different randomly selected flies per genotype highlighted sleep fragmentation in the *mbt^P1^* flies by the appearance of many more activity spikes compared to the other animals (Fig. 2K). Despite increased sleepiness at daytime, fragmented sleep inevitably led to higher values in the mean activity profile. Therefore, whereas *mbt^P1^* mutant flies displayed fragmented sleep, similar to PD patients, their total sleep at night and activity amount were not affected.

### Expression of Mbt in DA PAM cluster neurons is essential for normal climbing ability and life expectancy of flies

**Fig. 3.**
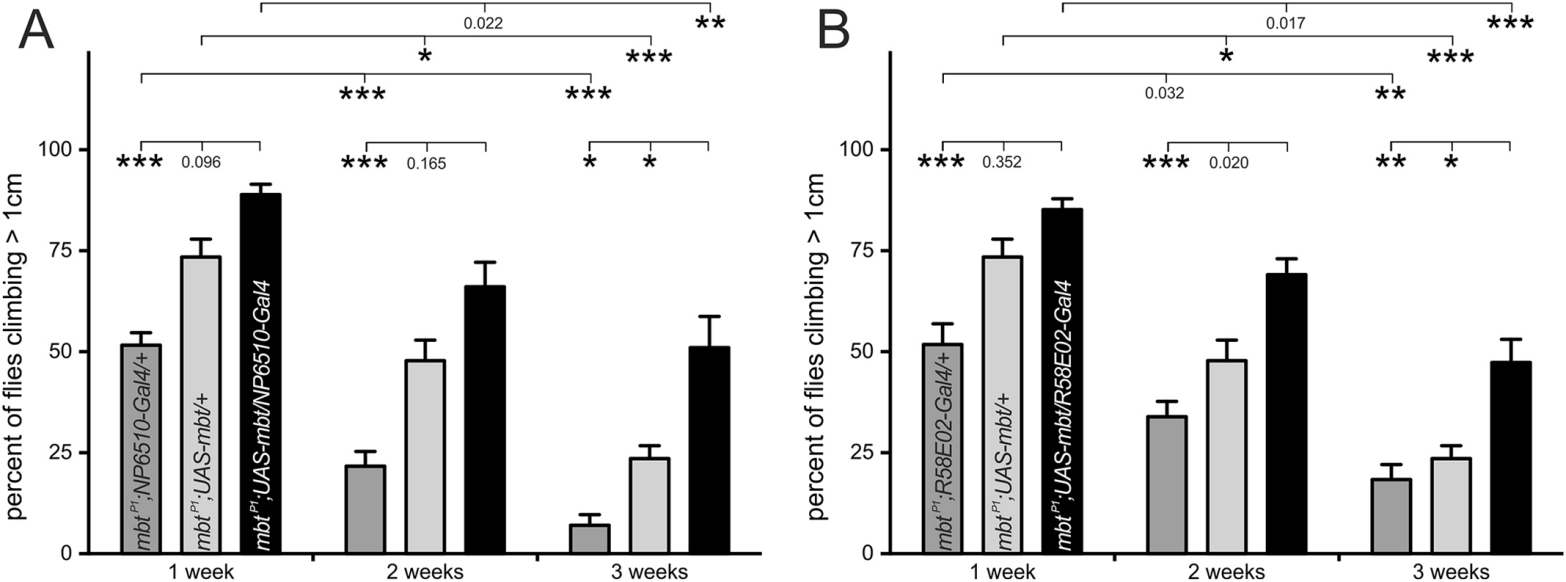
**Climbing ability of flies that re-express Mbt only in subsets of protocerebral anterior medial (PAM) neurons.** (A,B) Climbing performance within the first 3 weeks of life of the genotypes *mbt^P1^;NP6510-Gal4/UAS-mbt* (black in A) and *mbt^P1^;R58E02-Gal4/UAS-mbt* (black in B) compared to their controls *mbt^P1^;UAS-mbt/+* (light gray in A,B) and *mbt^P1^;NP6510-Gal4/+* (dark gray in A) or *mbt^P1^;R58E02-Gal4/+* (dark gray in B), respectively. Depicted are mean±s.e.m. of seven to 18 independent cohorts. *P<0.01, **P<0.001, ***P<0.0001 (Mann–Whitney test followed by Bonferroni correction).

A hallmark of PD is progressive loss or dysfunction of DA neurons. There are ∼300 DA neurons in the *Drosophila* brain, which are grouped into several bilateral arranged clusters localized anterior and posterior in the brain. Among these, the PAM cluster is the largest, comprising, depending on the study, from 100 to 134 DA neurons in each brain hemisphere (Liu et al., 2012; Mao and Davis, 2009). PAM neurons are involved in many processes such as memory formation, sleep-wake regulation and negative geotaxis (Kasture et al., 2018). Furthermore, a link between a small subgroup of ∼15 PAM neurons and climbing impairments in an α-synuclein PD model was established (Riemensperger et al., 2013). Based on these findings, we addressed the question whether expression of Mbt in this subgroup of PAM neurons is sufficient to restore startle-induced locomotion in the climbing assay. For that purpose, we used *NP6510-Gal4* (Aso et al., 2010; Riemensperger et al., 2013; Tanaka et al., 2008) to drive expression of a *UAS-mbt* transgene in this PAM subgroup in an otherwise *mbt^P1^* mutant background. *mbt^P1^;NP6510-Gal4/UAS-mbt* flies performed better than the corresponding control flies *mbt^P1^;UAS-mbt/+* and *mbt^P1^;NP6510-Gal4/+* (Fig. 3A). In particular, improvement was significant in 3-week-old animals. To substantiate these findings, we used the driver line *R58E02-Gal4*, which enabled expression of *UAS-mbt* in approximately three-quarters of PAM neurons including the NP6510 subgroup (Liu et al., 2012; Sun et al., 2018). Mbt expression in this larger set of PAM neurons restored the climbing ability of flies to a very similar degree to that of flies expressing *mbt* under *NP65110-Gal4* control (Fig. 3B). These data indicate a critical function of Mbt in NP6510 PAM neuron-dependent control of locomotor reactivity.

**Fig. 4.**
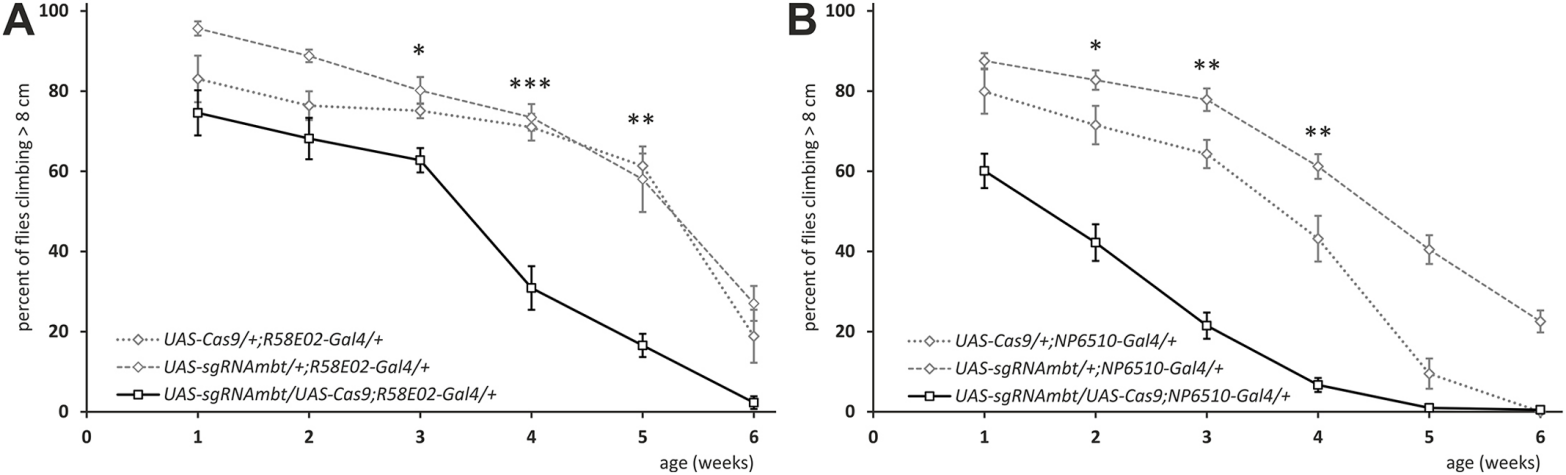
**Climbing ability of flies with *mbt* knockout in subsets of PAM neurons.** (A,B) Depicted is the ability to climb 8 cm or more within 10 s over a period of 6 weeks. In A, flies of the genotype *UAS-sgRNAmbt/UAS-Cas9;R58E02-Gal4/+* are compared to controls *UAS-sgRNAmbt/+;R58E02-Gal4/+* as well as *UAS-Cas9/+;R58E02-Gal4/+*. In B, flies of the genotype *UAS-sgRNAmbt/UAS-Cas9;NP6510-Gal4/+* are compared to *UAS-sgRNAmbt/+;NP6510-Gal4/+* as well as *UAS-Cas9/+;NP6510-Gal4/+*. Shown are mean±s.e.m. of seven to 12 independent cohorts, with the exception of genotype *UAS-Cas9/+;R58E02-Gal4/+* in week 6 with only five cohorts. The *P*-value is at least as depicted for each time point. *P<0.01, **P<0.001, ***P<0.0001 (Mann–Whitney test followed by Bonferroni correction).

It should be noted that the rescue was incomplete in both cases (compare Fig. 3A,B with Fig. 1A) and was mainly confined to attenuate the progressive, age-dependent phenotype. One explanation could be inappropriate expression levels of the *mbt* transgene. A second possibility could be a function of Mbt in developmental processes, which is not covered by *R58E02-Gal4*- or *NP6510-Gal4*-driven expression of Mbt in differentiated neurons (see below). A third possibility considers the additional requirement of Mbt function outside of PAM cluster neurons to accomplish full climbing performance. *NP6510-Gal4*-expressing PAM neurons innervate the mushroom bodies (Riemensperger et al., 2013), a paired brain structure involved in olfactory learning processes (Cognigni et al., 2018), but also in locomotion control (Martin et al., 1998; Serway et al., 2009; Sun et al., 2018). Because *mbt^P1^* flies have a reduced number of mushroom body neurons (Melzer et al., 2013; Melzig et al., 1998), this might limit climbing performance in *mbt^P1^;NP6510-Gal4/UAS-mbt* flies. To specifically look at Mbt function in PAM neurons, we aimed to eliminate Mbt using cell-type-specific CRISPR/Cas9 (Port et al., 2020). First, we validated the specificity of the CRISPR/Cas9 *mbt* knockout by inducing known *mbt* mutant phenotypes with tissue-specific *Gal4* driver lines, including rough eyes (Fig. S2A, Table S1) and tiny mushroom bodies (Fig. S2B, Table S1) in the adult with *ey^OK107^-Gal4* as well as absence of Mbt expression in the larval eye imaginal disc with *DE-Gal4* (Fig. S2C, Table S1). Based on this verification, the influence of the *mbt* knockout in PAM neurons on climbing performance of *UAS-sgRNAmbt/UAS-Cas9;R58E02-Gal4/+* flies was analyzed and compared to the controls *UAS-sgRNAmbt/+;R58E02-Gal4/+* and *UAS-Cas9/+;R58E02-Gal4/+*. Young flies (until week 3) of all genotypes climbed well, with no significant differences in their ability to reach the threshold of 1 cm height (Fig. S3A). This was strikingly different to the poor climbing ability of young *mbt^P1^* flies (Fig. 1A). In order to recognize weaker effects on climbing performance, we chose a higher threshold (> 8 cm). This type of analysis revealed a slight, but again not a significant, decrease in the climbing ability of the experimental group compared to the controls in the first 2 weeks. However, differences became evident with increasing age. Although performance of all genotypes reduced with age, the climbing ability of *UAS-sgRNAmbt/UAS-Cas9;R58E02-Gal4/+* flies declined significantly faster starting in week 3 and strongly dropped in week 4 (Fig. 4A). To substantiate these results and to check whether removal of Mbt in the NP6510 subgroup of PAM neurons is decisive, we used the *NP6510-Gal4* driver line for CRISPR/Cas9-mediated knockout of *mbt* and observed a decline in climbing performance using the 1 cm (Fig. S3B) and 8 cm (Fig. 4B) thresholds. The targeted *mbt* knockout in NP6510 neurons caused an earlier effect on climbing. In summary, Mbt is required in the NP6510-marked subgroup of PAM neurons as part of the neural circuitry controlling startle-induced locomotion, and removal of Mbt in these neurons is associated with age-dependent, progressive impairment of this behavior.

**Fig. 5.**
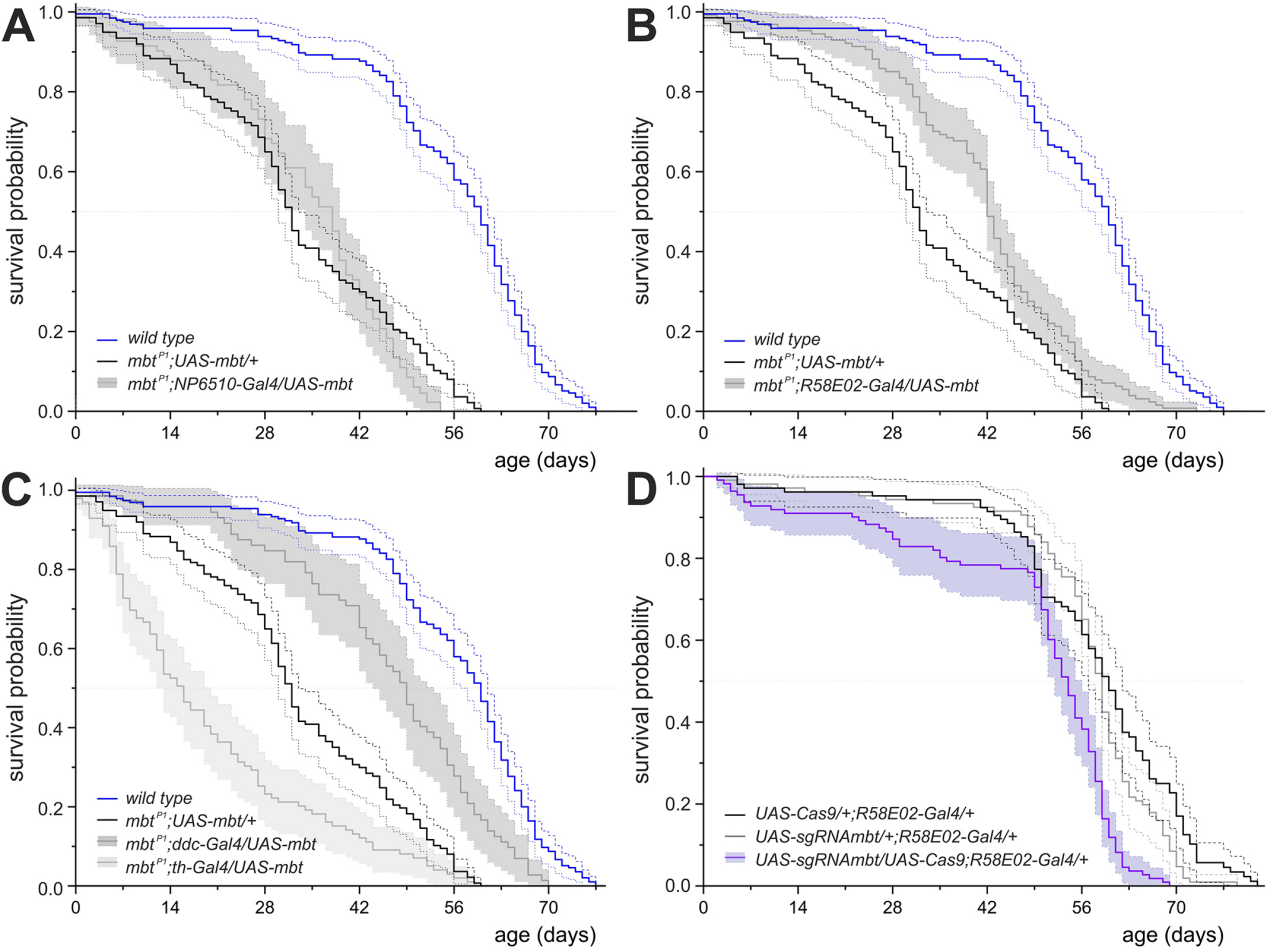
**Lifespan analysis of flies re-expressing Mbt in subsets of dopaminergic (DA) neurons or with *mbt* knockout in R58E02 cells.** (A-C) Depicted is the survival probability, including the 95% confidence intervals, of the genotypes *mbt^P1^;NP6510-Gal4/UAS-mbt* (gray in A, *n*=82), *mbt^P1^;R58E02-Gal4/UAS-mbt* (gray in B, *n*=127) as well as *mbt^P1^;Ddc-Gal4/+;UAS-mbt/+* (dark gray in C, *n*=72) and *mbt^P1^;th-Gal4/UAS-mbt* (light gray in C, *n*=99). For comparison, the graphs (A-C) include the survival curves of the controls *mbt^P1^;UAS-mbt/+* (black in A-C, *n*=137) and wild type (blue in A-C, *n*=195). The survival probabilities of the *mbt^P1^;Gal4* controls are shown in Fig. S4. Significant differences compared to *mbt^P1^;UAS-mbt/+* and the corresponding Gal4 control can be observed for *mbt^P1^;Ddc-Gal4/+;UAS-mbt/+* (*P*<0.0001), *mbt^P1^;th-Gal4/UAS-mbt* (*P*<0.0001) and *mbt^P1^;R58E02-Gal4/UAS-mbt* (*P*<0.001). *mbt^P1^;NP6510-Gal4/UAS-mbt* flies are not significantly different from their controls. (D) *UAS-sgRNAmbt/UAS-Cas9;R58E02-Gal4/+* flies (violet, *n*=111) live significantly shorter than controls *UAS-sgRNAmbt/+;R58E02-Gal4/+* (*P*<0.0001, gray in D, *n*=106) and *UAS-Cas9/+;R58E02-Gal4/+* (*P*<0.0001, black in D, *n*=105). Statistical analysis of the survival data was performed using Kaplan–Meier analysis and the log-rank test followed by Bonferroni correction. Details on the results of the statistical evaluation are in Fig. S4.

As in the case of *mbt^P1^*, premature progressive decline in climbing abilities often, but not always, correlates with decreased lifespan in other *Drosophila* PD models (Basil et al., 2017; Butler et al., 2012; Haywood and Staveley, 2004; Lavara-Culebras and Paricio, 2007; Park et al., 2006; Poddighe et al., 2013), implicating at least some mechanistic or cellular relationships. We therefore asked whether PAM neuron-driven expression of *UAS-mbt* with *NP6510-Gal4* or *R58E02-Gal4* in an *mbt^P1^* background restored normal life expectancy. Expression of Mbt only in the *NP6510-Gal4*-positive PAM neurons was not sufficient to improve lifespan (Fig. 5A; Fig. S4). However, the lifespan of *mbt^P1^;R58E02-Gal4/UAS-mbt* animals had increased, but not to that of the wild-type animals (Fig. 5B; Fig. S4).

To verify a positive influence of Mbt expression in PAM neurons on survival, we repeated the experiments with the DA neuron driver lines *th-Gal4* (Friggi-Grelin et al., 2003) and *Ddc-Gal4* (Li et al., 2000). *th-Gal4* allows for the expression of transgenes in all DA neurons except in the PAM cluster, where expression is restricted to a small fraction of ∼13 neurons distinct from the NP6510 subgroup. Expression with *Ddc-Gal4* targets the majority of PAM neurons, but not all other DA cell clusters, and, in addition, many serotonergic neurons (Aso et al., 2010; Claridge-Chang et al., 2009; Liu et al., 2012; Mao and Davis, 2009; Pech et al., 2013; Sun et al., 2018). Animals of the genotype *mbt^P1^;Ddc-Gal4/+;UAS-mbt/+* reached slightly older age than *mbt^P1^;R58E02-Gal4/+;UAS-mbt/+* flies (Fig. 5C; Fig. S4). This difference might be an indication that Mbt expression in serotonergic neurons also has an influence on life expectancy, as serotonin signaling has an impact on lifespan (Chakraborty et al., 2019; Ro et al., 2016). In combination with the *R58E02-Gal4* experiment, the result with the *mbt^P1^;Ddc-Gal4/+;UAS-mbt/+* flies supports the idea that Mbt expression in PAM neurons increases life expectancy. In accordance with this hypothesis, *th-Gal4*-driven expression of Mbt did not improve lifespan but even reduced it (Fig. 5C; Fig. S4), a phenomenon that we cannot explain at the moment.

Although life expectancy increased upon Mbt re-expression in PAM neurons, wild-type lifespan was not reached, which might be explained by the organismic *mbt* null background. To investigate whether premature animal death can be induced by targeted *mbt* knockout in PAM neurons, we used R58E02 cell-type-specific CRISPR/Cas9 (Port et al., 2020). Compared to the controls *UAS-sgRNAmbt/+;R58E02-Gal4/+* and *UAS-Cas9/+;R58E02-Gal4/+*, the *UAS-sgRNAmbt/UAS-Cas9;R58E02-Gal4/+* flies have a significantly shorter life expectancy. These animals showed a slightly increased probability of death at the beginning of life, which then increased from week 8 onwards (Fig. 5D). The maximal life expectancy of these flies was 68 days – more than 10 days shorter than that of wild-type flies (Fig. 1B).

In summary, climbing ability and life expectancy have an unequal dependency of Mbt expression in PAM neurons. In the case of climbing ability, Mbt expression only in NP6510 PAM neurons is an important prerequisite to protect against a premature loss of climbing performance. Modifying Mbt function in additional PAM cells, either by knockdown or re-expression, does not enhance the observed effects. In contrast, a long fly life is achieved only by broader expression of Mbt in PAM neurons.

### Loss of Mbt leads to a developmental reduction in PAM neurons but has no obvious impact on neurodegeneration

As shown, the strong climbing impairment of young *mbt^P1^* flies (Fig. 1A) was neither phenocopied by deletion of Mbt in PAM neurons (Fig. 4) nor completely rescued by re-expression of Mbt in PAM neurons of *mbt^P1^* mutant flies (Fig. 3). Our consideration was that this early phenotype is not a consequence of dysfunction of differentiated PAM neurons, but could be due to a developmental defect in the generation of PAM neurons. Previous studies implicated Mbt in the proliferation control of at least one subgroup of neural progenitor cells [neuroblasts (NBs)] in the developing *Drosophila* brain, the mushroom body NBs (Melzer et al., 2013), leaving open the question of a more general requirement of Mbt for normal NB proliferation.

**Fig. 6.**
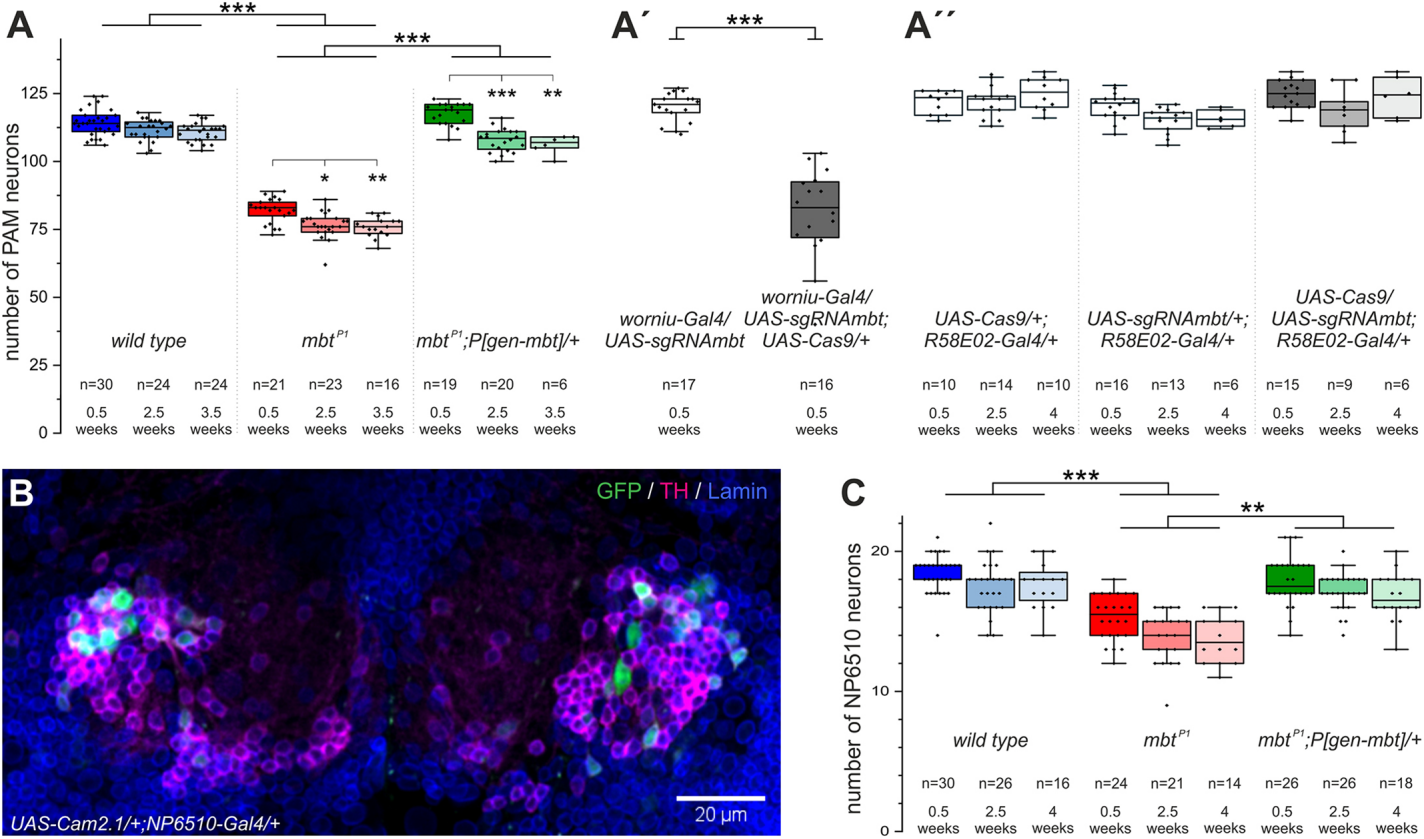
**Effects of organismic or cell-type-specific *mbt* knockout on adult PAM neuron numbers.** Quantification of PAM cluster neurons. (A) PAM neuron numbers per brain hemisphere of wild-type, *mbt^P1^* and *mbt^P1^;P[gen-mbt]/+* flies at three different ages. (A′) Neuroblast-specific *mbt* knockout in *worniu-Gal4/UAS-sgRNAmbt;UAS-Cas9/+* flies significantly reduces the PAM cell number compared to the control *worniu-Gal4/UAS-sgRNAmbt.* (A′′) PAM neuron-specific *mbt* knockout in *UAS-Cas9/UAS-sgRNAmbt;R58E02-Gal4/+* flies has no effect on cell number at different ages compared to the controls *UAS-Cas9/+;R58E02-Gal4/+* and *UAS-sgRNAmbt/+;R58E02-Gal4/+.* (B) Confocal image of PAM cluster neurons. For the analyses shown in A-A′′, fly brains were stained with anti-TH to label DA cells (magenta) and anti-Lamin (blue) as a nuclear membrane marker. For counting *NP6510-Gal4* expressing PAM neurons (C), brains were additionally stained with an anti-GFP antibody to visualize *UAS-Cam2.1* expression as seen in B. (C) Number of NP6510 TH-positive neurons within the PAM cluster per brain hemisphere in wild-type, *mbt^P1^* and *mbt^P1^;P[gen-mbt]/+* animals at three different ages. Sample sizes are indicated on the graphs. The box plots indicate the median and quartile values; the whiskers are drawn up to a maximum of 1.5 times the interquartile range or the respective minimum/maximum values. Outliers are indicated. **P<0.001, ***P<0.0001 (Mann–Whitney test followed by Bonferroni correction). Highlighted are significant differences within one genotype at different time points and significant differences between different genotypes at the same time point. For the latter analysis, at least the specified level of significance is reached at all time points.

To analyze whether the number of PAM cluster neurons is affected already in young *mbt^P1^* flies, we labeled DA neurons using an antibody against Tyrosine hydroxylase (TH; also known as Ple), the rate-limiting enzyme in dopamine synthesis. Co-staining for the nuclear membrane protein Lamin allowed us to distinguish the densely packed DA neurons in the PAM cluster and to unambiguously identify cells with only weak TH expression. In young (0.5 weeks old) *mbt^P1^* animals, the number of PAM neurons was only 70% of the wild-type value, a phenotype that was completely reverted in the presence of *P[gen-mbt]* (Fig. 6A)*.* The clear reduction in PAM cluster neurons in young *mbt^P1^* flies strongly argues for a developmental problem. To substantiate this finding, we performed *mbt* knockout in NBs using the NB-specific driver *worniu-Gal4*. Whereas the PAM cell number in *UAS-sgRNAmbt/worniu-Gal4* controls corresponded to that of wild type, the *UAS-sgRNAmbt/worniu-Gal4;UAS-Cas9/+* flies had significantly fewer PAM cells (Fig. 6A′), very similar to *mbt^P1^* flies (Fig. 6A). Because the *worniu-Gal4* expression pattern includes also mushroom body NBs, we observed tiny mushroom bodies in *UAS-sgRNAmbt/worniu-Gal4;UAS-Cas9/+* flies (Table S1; T.R. and S.M.P., unpublished observation), like in the case of *UAS-sgRNAmbt/+;UAS-Cas9/+;eyOK107-Gal4/+* (Fig. S2B). Although specific labeling and evaluation of NBs generating adult PAM neurons [CREa1 and CREa2 (Lee et al., 2020)] was not possible due to lack of suitable reagents, these findings argue for a NB proliferation defect as the cause of the reduced number of PAM neurons in young *mbt^P1^* animals. It also provides an explanation as to why young *mbt^P1^* flies show such poor climbing performance.

**Fig. 7.**
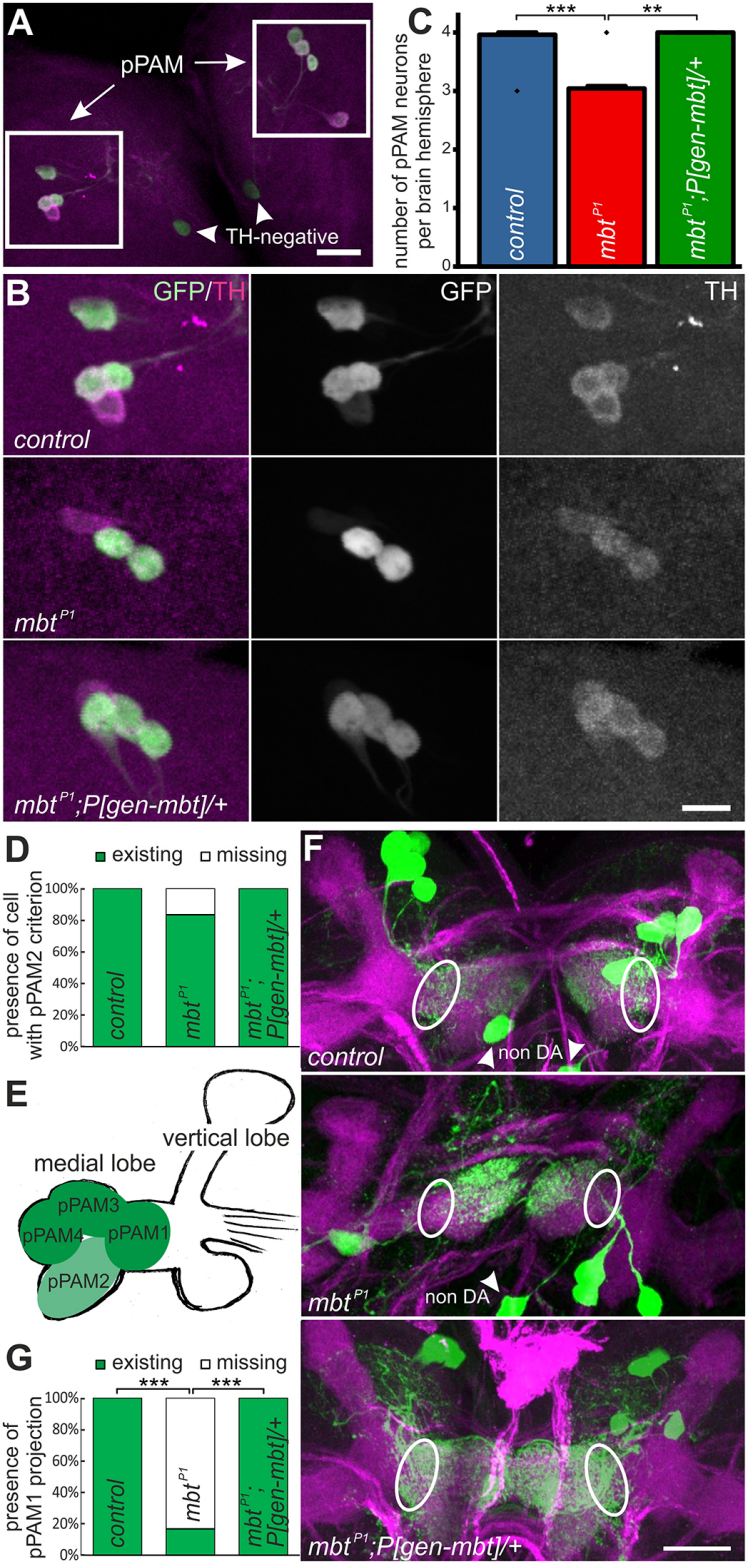
**Presence of primary (p)PAM** **neurons in larvae.** (A) A section from a larval brain including the pPAM neurons of the genotype *UAS-Cam2.1/+;R58E02-Gal4/+.* Scale bar: 20 µm. For the analysis shown in B and C, larval brains were stained with anti-TH to label DA cells (magenta) and co-stained with an anti-GFP antibody to visualize *UAS-Cam2.1* expression (green) driven by *R58E02-Gal4* to highlight pPAM cells in all investigated genotypes. (B) Third instar larval pPAM clusters of *mbt^P1^* animals compared to control and *mbt^P1^;P[gen-mbt]/+*. Scale bar: 10 µm. (C) Number of pPAM neurons in control (*n*=26), *mbt^P1^* (*n*=24) and *mbt^P1^;P[gen-mbt]/+* (*n*=4) larvae. (D) Quantification of pPAM2 presence in brain hemispheres from control (*n*=24), *mbt^P1^* (*n*=24) and *mbt^P1^;P[gen-mbt]/+* (*n*=4). (E) Schematic representation of the larval mushroom body and innervation of the medial lobe by pPAM neurons. (F) Confocal images of *UAS-Cam2.1/+;R58E02-Gal4/+*-expressing pPAM neuron projections (green) on the medial lobe of the larval mushroom body stained with Fas2 (magenta). The projection of pPAM1 (encircled) is missing in *mbt^P1^* brains compared to control and *mbt^P1^;P[gen-mbt]/+*. The *UAS-Cam2.1/+;R58E02-Gal4/+*-positive but non-DA neurons are indicated (arrowheads). Scale bar: 20 µm. (G) Quantification of pPAM1 presence in brain hemispheres from control (*n*=26), *mbt^P1^* (*n*=24) and *mbt^P1^;P[gen-mbt]/+* (*n*=18) larvae. **P<0.001, ***P<0.0001 (Mann–Whitney test followed by Bonferroni correction).

To examine the influence of Mbt on the generation of DA neurons in more detail, we extended the analysis to the four larval primary (p)PAM neurons (Rohwedder et al., 2016). Using the *R58E02-Gal4* driver line for labeling pPAM neurons with a fluorescent construct, it became evident that the number of four pPAM cells in each brain hemisphere of control and *mbt^P1^;P[gen-mbt]/+* larvae was reduced in nearly all cases to three cells in *mbt^P1^* (Fig. 7A-C).

This result raised the question as to whether always the same pPAM neuron is missing in *mbt^P1^* larvae. The pPAM2 neuron weakly expresses the *R58E02*-driven fluorescent marker (Rohwedder et al., 2016). A cell with these characteristics was absent in ∼15% of larval brain hemispheres of *mbt^P1^* (Fig. 7D). To distinguish pPAM1, pPAM3 and pPAM4, we followed their differential projections to distinct zones of the medial axonal lobe of the larval mushroom body stained with Fas2 (Fig. 7E,F; Rohwedder et al., 2016). In more than 80% of cases, we observed loss of the pPAM1 projections in *mbt^P1^* brain hemispheres (Fig. 7G), indicating loss of this cell, whereas pPAM3 and pPAM4 neurons were not affected. This finding leaves open the interesting question whether the reduced number of PAM neurons in adult *mbt^P1^* brains might be caused by a failure in the generation of specific PAM subclasses. The large number of PAM neurons, their subdivision into multiple subgroups and the complexity of their mushroom body innervation patterns (Lee et al., 2020; Li et al., 2020) provide major challenges for this analysis.

Considering the early PAM neuron deficit in *mbt^P1^*, the question arises whether the age-dependent progressive decline in climbing performance and the shortened life expectancy correlates with a corresponding further loss of PAM neurons. DA neuron loss is a neuropathological hallmark of PD; however, in different *Drosophila* PD models, progressive loss of DA neurons was not consistently observed (Botella et al., 2009; Navarro et al., 2014). As a first indirect parameter, we quantified TH protein levels by western blot analysis of head lysates. Several blots indicated a slight reduction in the TH levels of 0.5-week-old *mbt^P1^* head extracts compared to those of controls, which could reflect the reduction in PAM neurons in *mbt^P1^* (Fig. S5A,B). A more pronounced reduction was seen in 3-week-old *mbt^P1^* animals (Fig. S5C), indicating either a further reduction in DA neuron number or an age-dependent functional impairment of DA neurons.

Based on this finding, we counted PAM neurons in the brains of 2.5- and 3.5-week-old animals. In comparison to young *mbt^P1^* and control animals, a slight age-dependent decrease in PAM neurons was observed in all genotypes (Fig. 6A). Decrease was more pronounced between 0.5- and 2.5-week-old animals, but then cell numbers remained rather constant until the last week of measurement (Fig. 6A). This early effect may reflect fine adjustment of neural circuitry during the first phase of adult life. We concluded that, at the level of the whole PAM cluster, Mbt is required to form the normal number of neurons during development, but the neurons’ survival during adulthood is not or at least largely independent of Mbt function. This conclusion was also supported by the staining of brains from 2-week-old animals for the apoptotic cell death marker Dcp-1, which provided no hint for apoptotic PAM neurons in all genotypes (T.R., unpublished observation).

Because of their manifold relationships to behaviors such as sleep and locomotion control, we extended our analysis also to the protocerebral posterior lateral (PPL)1, PPL2, protocerebral anterior lateral (PAL), protocerebral posterior medial (PPM)1/2 and PPM3 clusters in the brain, each of which only contains a few DA neurons (Kasture et al., 2018). We could not observe any significant deviation in cell numbers between wild-type, *mbt^P1^* and *mbt^P1^;P[gen-mbt]/+* animals at the age of 0.5 and 3 weeks (S.M.P., unpublished observation).

In order to confirm that loss of Mbt function has no cell-autonomous effect on survival of differentiated PAM neurons, we chose an experimental approach in which the developmental aspect and neurodegeneration can be separated from each other. The cell-type-specific knockout of *mbt* in the majority of PAM neurons in *UAS-sgRNAmbt/UAS-Cas9;R58E02-Gal4/+* animals did not result in changes in overall PAM neuron number compared to the controls in young flies, and did not induce neurodegeneration in 2.5- and 4-week-old animals (Fig. 6A″). Thus, loss of Mbt in differentiated PAM neurons has no significant impact on their survival. This experiment also supported our conclusion that the reduced number of PAM cells in *mbt^P1^* animals (Fig. 6A) can be attributed to a developmental defect (Fig. 6A′).

However, the rather high variability in the number of PAM neurons in different animals of the same genotype might mask minor effects of Mbt on the small NP6510 subpopulation of DA neurons required for startle-induced climbing. Co-stainings for TH, Lamin and *NP6510-Gal4-*driven fluorescent protein (Fig. 6B) in 0.5-, 2.5*-* and 4*-*week*-*old animals confirmed a reduced cell number in *mbt^P1^* flies and again showed a slight, age-dependent decrease in cell numbers for all genotypes at the time point of 2.5 weeks (Fig. 6C), similar to the whole PAM cluster analysis (Fig. 6A). This again indicates that loss of Mbt does not cause neurodegeneration.

Based on our results, we can make two statements. First, an influence of Mbt on neurodegenerative cell loss is highly unlikely. Rather, an Mbt-dependent functional impairment of the PAM cells can be assumed. Second, the reduced number of PAMs in young *mbt^P1^* has a developmental cause. The impairment in climbing ability already seen in young *mbt^P1^* animals correlates with a reduced number of PAM neurons, but the progressive, age-dependent decline in climbing performance in *mbt^P1^* and upon NP6510 neuron-specific *mbt* knockout is not reflected by a corresponding decrease in PAM neurons.

## DISCUSSION

In this study, we demonstrated that, in the absence of Mbt, flies show a wide range of phenotypes related to PD. For the first time, we were able to show that Mbt is required to establish and maintain normal motor skills. *mbt^P1^* flies have very bad motor skills from the time point of eclosion, which worsened significantly with age (Fig. 1A), a key criterion for PD. In addition, *mbt^P1^* flies meet other PD criteria including reduced life expectancy (Fig. 1B), higher immobility values in the FST (Fig. 1C) and sleep impairment (Fig. 2). Although independent of age, loss of Mbt function has a negative effect on the number of DA PAM neurons (Fig. 6A) as part of the neural circuity controlling motor behavior. Similar to the diagnosis of PD in humans, which is based on a variety of clinical criteria (Jankovic, 2008; Kalia and Lang, 2015; Poewe et al., 2017), the sum of the phenotypes we observed allows the conclusion that *mbt^P1^* animals show PD-like behavior.

Frequently, the question arises whether *Drosophila* is a good model organism to recapitulate the complexity of PD phenotypes, a question also valid for other model organisms (Breger and Fuzzati Armentero, 2019; Dung and Thao, 2018; Taguchi et al., 2020). One should bear in mind that the symptomatology of PD is heterogeneous and not every patient suffers from all symptoms (Kalia and Lang, 2015; Obeso et al., 2010; Poewe et al., 2017). The true face of PD can only be understood by correlating the variability of the various symptoms with the respective trigger of the disease (e.g. gene mutations in the individual genetic background, toxins and living conditions). It is therefore not surprising that none of the vertebrate PD models is able to completely reflect all aspects of PD (Breger and Fuzzati Armentero, 2019; Taguchi et al., 2020). Nevertheless, in *Drosophila*, very complex PD-like phenotypes and behaviors can be induced by mutations, e.g. in *Pink1*, including reduced life expectancy (Poddighe et al., 2013), decreasing motor skills (Park et al., 2006), sleep fragmentation (Valadas et al., 2018), olfactory dysfunctions (Poddighe et al., 2013), cognitive impairments (Julienne et al., 2017) and DA cell loss (Wang et al., 2006). Looking at our checklist elaborated from many *Drosophila* PD studies (Table 1) in relation to characterization of potential PD candidate genes such as *mbt*, the requirement to extend the analysis to more than just one symptom becomes evident. Motor skill impairment can have different reasons, but the combination with other cellular and/or behavioral deficits points to a PD-like phenotype.

Adaptation of mammalian paradigms such as the OFT and the FST allows the investigation of emotional behaviors in flies also in relation to PD. Paraquat administration as an oxidative stress inducer caused depression-like behavior (Neckameyer and Nieto-Romero, 2015), and α-synuclein expression resulted in increased centrophobism indicative of enhanced anxiety (Chen et al., 2014a). Changes in emotional behavior can also be observed as a consequence of mutation in *mbt*. Whereas *mbt^P1^* flies behaved inconspicuously in the OFT (Fig. S1), they remained more immobile in the FST (Fig. 1C). Depending on study, increased immobility time is interpreted as a depression-like state, bad mood or an impaired ability to cope with an acute inescapable stressor (Commons et al., 2017; Molendijk and de Kloet, 2019). However, reduced motor skills as observed for *mbt^P1^* in the climbing assay could affect immobility time in the FST. One should also consider the possibility that motor skills and emotional state may influence each other. Because spontaneous activity is not impaired in *mbt^P1^* animals (Fig. 2A), the inner drive for movement is apparently maintained. However, under a stressful situation in the FST, they respond with decreased mobility. This can be interpreted as a direct consequence of impaired stress coping. Alternatively, flies realized quickly that their impaired motor skills do not allow escaping from this unpleasant situation. The switch to a more passive behavior favors survival until a new escape option might appear. At the moment, we cannot distinguish between these possibilities.

Sleep disorders are one of the most frequent symptoms observed already in early phases of PD (Chaudhuri et al., 2006; Högl et al., 2018; Jankovic, 2008; Kalia and Lang, 2015). Recapitulating sleep disturbances even in rodent PD models remains a challenging task because of the complexity of this behavior and species-specific differences in sleep architecture (Medeiros et al., 2019). *Drosophila* fulfills criteria for sleep, including a specific sleep posture, consolidated periods of immobility, increased arousal threshold during these periods, a significant sleep rebound after sleep deprivation and modulation of electrical brain activity in relation to the activity state of the fly (Helfrich-Förster, 2018). Detailed sleep analysis in *Drosophila* PD models, to our knowledge, was so far only carried out for Pink1 and Parkin (Valadas et al., 2018). In this study, *parkin* and *Pink1* null mutant flies showed pronounced sleep fragmentation, a phenotype also reported for PD patients. In addition, *parkin* and *Pink1* flies showed diminished morning anticipation (Valadas et al., 2018). In *mbt^P1^* flies, we also observed changes in these sleep parameters. Although the influence of *mbt^P1^* on morning anticipation was not pronounced (Fig. 2H), there are clear parallels to the finding with *parkin* and *Pink1* regarding sleep fragmentation (Fig. 2I-K). Furthermore, we noticed increased sleepiness during the day in *mbt^P1^* animals (Fig. 2G), a symptom in PD patients (Jankovic, 2008; Kalia and Lang, 2015; Poewe et al., 2017) not observed previously in *Drosophila* PD models. The cellular origin of these phenotypes in *mbt^P1^* flies is unknown. However, looking at knockdown of *parkin* and *Pink1* in specific neuron subpopulations, some assumptions can be made. RNA interference of either gene in a subgroup of clock neurons, the ventral lateral neurons (LN_v_s), prevented anticipation of dawn (Valadas et al., 2018). In wild type, the small LN_v_s signal via DA PPM3 neurons to the ellipsoid body to drive initiation of pre-dawn locomotor activity (Liang et al., 2019). However, sleep fragmentation was observed upon knockdown in insulin-producing cells (IPCs) and, at least in case of *parkin*, also in DA neurons. These phenotypes correlated with accumulation of neuropeptides [Insulin-like peptide type 2 (Ilp2) in IPCs, pigment-dispersing factor (Pdf) in LN_v_s] in the cell bodies, and was accompanied by reduced vesicular transport to their terminal release sites (Valadas et al., 2018). Serotonergic neurons can also have an influence on sleep fragmentation in *Drosophila*. Increased serotonin release leads to fragmented sleep by transmitting signals through the 5HT7 receptor to the ellipsoidal body, but does not affect total sleep (Liu et al., 2019). These findings underline the importance of different neuropeptidergic neurons to explain sleep disturbances as one major non-motor PD symptom and support the view that non-DA neurons have a significant impact on the complex behavioral disturbances in PD (Schapira et al., 2017).

Our data assign a critical function of Mbt in DA PAM neurons. PAM cells contribute to a wide range of functions including memory formation, negative geotaxis, regulation of the sleep-wake cycle, foraging and food intake (Kasture et al., 2018; Landayan and Wolf, 2015). The PAM cluster is not a homogeneous cell group, which is reflected by the differential expression patterns of the Gal4 drivers TH, NP6510 and R58E02 (Liu et al., 2012; Pech et al., 2013; Riemensperger et al., 2013; Sun et al., 2018). Our finding that life expectancy was improved upon Mbt re-expression in the large R58E02-positive cell population (Fig. 5B), whereas Mbt expression in the small NP6510 population is sufficient to protect flies from premature loss of their climbing ability (Figs. 3A and 4B; Fig. S3B), provided further evidence for the functional heterogeneity of PAM cells (Nagoshi, 2018). This heterogeneity is reminiscent of the diversity of DA neurons in the SNpc of humans (Vogt Weisenhorn et al., 2016). *Drosophila* PD models established the importance of the PAM cluster neurons to maintain age-appropriate climbing performance and pinpointed this function to the NP6510 PAM subgroup (Bou Dib et al., 2014; Riemensperger et al., 2013; Sun et al., 2018; Tas et al., 2018). Using targeted expression or knockdown, we have now provided evidence that Mbt is a critical molecular player in NP6510 PAM neurons to maintain climbing ability. In vertebrates, oligomeric α-synuclein inhibits the Mbt homolog PAK4, and PAK4 activity is relevant for motor skills (Danzer et al., 2007; Won et al., 2016). Because *Drosophila* has no α-synuclein homolog, such a regulatory mechanism can be excluded in the case of wild-type flies. Yet, progressive climbing deficits can be induced by ectopic expression of a mutant form of human α-synuclein in NP6510 PAM neurons (Riemensperger et al., 2013). Therefore, it might be interesting to determine whether progressive loss of climbing performance upon human α-synuclein expression in flies is caused by a negative impact on Mbt signaling to (the so far unknown) downstream targets.

Normal life expectancy of *Drosophila* depends on many factors such as oxidative stress, Serotonin, Insulin and TOR signaling, calorie intake and dietary restriction (Chakraborty et al., 2019; Hwangbo et al., 2004; Kapahi et al., 2017; Partridge et al., 2011; Ristow and Zarse, 2010; Ro et al., 2016). An influence on life expectancy is also discussed for individual brain regions and cell clusters such as the fat body or DA neurons (Hwangbo et al., 2004; Tas et al., 2018; Trostnikov et al., 2020; Xiaolin, 2020 preprint). Interestingly, there seem to be overlapping but not identical mechanisms controlling life expectancy and age-related decline in locomotor ability (Jones and Grotewiel, 2011). This fits with our observations: expression of Mbt in the NP6510 PAM subpopulation relevant for climbing does not prolong lifespan in an otherwise *mbt* mutant background (Fig. 5A), but a broader expression of Mbt in PAM neurons using *R58E02-Gal4* or *Ddc-Gal4* increased lifespan (Fig. 5B,C). Conversely, selective Mbt knockout in *R58E02*-expressing PAM neurons reduced life expectancy (Fig. 5D). Because the R58E02 PAM neurons include the NP6510 cells, normal lifespan is either dependent on Mbt expression in the majority of PAM neurons, which might also include NP6510 cells, or in a small cell population distinct from NP6510 PAM cells. Although we cannot completely rule out the possibility that the *R58E02-Gal4* and *Ddc-Gal4* expression patterns have some overlap elsewhere in the body, our findings support a role of Mbt in PAM neurons to reach normal life expectancy.

Because we linked reduced lifespan and age-dependent climbing impairment to loss of Mbt function in PAM neurons, a central question was whether these effects correlate with neurodegeneration of PAM cells. Although considered as a hallmark of PD, loss of DA neurons was not consistently observed in different *Drosophila* PD models (Botella et al., 2009; Navarro et al., 2014). For *mbt*, we made two important observations. First, PAM neuron number was already significantly reduced in young *mbt^P1^* flies (Fig. 6A). This phenotype provides a good explanation for the impaired climbing ability from the beginning of adult life (Fig. 1A). Second, the strong, age-dependent decline in climbing performance of *mbt^P1^* flies is not reflected by a further loss of PAM neurons (Figs. 1A and 6A). The minor reduction in PAM neurons in older *mbt^P1^* flies was also observed in control animals. This result was confirmed by CRISPR/Cas9-mediated knockout of *mbt* specifically in PAM neurons using the *R58E02-Gal4* driver line, again showing no effect on cell number (Fig. 6A″). This driver line is expressed in PAM and pPAM neurons starting from their generation during larval and pupal development (Agrawal and Hasan, 2015; Rohwedder et al., 2016). Thus, loss of Mbt has no cell-autonomous effect on PAM neuron survival during development and does not induce an apparent neurodegenerative effect during adult life, but strongly impairs PAM neuron function during aging. This is in marked contrast to what has been observed for the Mbt homolog PAK4 in vertebrates. Analysis of postmortem PD patients showed less PAK4 activity in apoptotic DA neurons, and activated PAK4 protected from neurotoxicity via phosphorylation of the CREB co-activator CRTC1 in rat models of PD (Won et al., 2016). Because the corresponding phosphorylation site in *Drosophila* CRTC isoforms is absent (sequence comparison in Fig. S6), we exclude a similar neuroprotective function of Mbt at least via the CRTC1–CREB axis.

Our finding that knockout of *mbt* in PAM neurons had no effect on cell number (Fig. 6A″), whereas the knockout already at the level of neural progenitor cells (NBs) caused PAM neuron loss to a very similar degree to that in *mbt^P1^* flies (Fig. 6A,A′), strongly argues for a proliferation defect of NB as the major cause. This correlates with previous results demonstrating impaired mushroom body NB proliferation as the cause of the *mbt^P1^* small mushroom body phenotype in the adult brain (Melzer et al., 2013). Supporting this conclusion, we recapitulated the mushroom body phenotype by CRISPR/Cas9-mediated knockout of *mbt* using two different driver lines (*worniu-Gal4* in all NBs, *ey^OK107^-Gal4* amongst others in mushroom body NBs). Mushroom body NBs are exceptional because they have an extended proliferation period far into pupal stage (Ito and Hotta, 1992) and sequentially generate distinct classes of neurons (Lee et al., 1999). In *mbt^P1^* animals, there is a pronounced lack of the latest born subclass of mushroom body neurons, suggesting a requirement for Mbt to maintain the proliferation capacity of NBs throughout development. Although other adult brain structures are not visibly affected in *mbt^P1^* flies (Melzig et al., 1998), the expression of Mbt in many brain NBs suggested a more common function (Melzer et al., 2013). The CREa1 and CREa2 NBs were recently identified as the progenitor cells for PAM neurons. They sequentially generate multiple classes of PAM neurons until early pupal stage with distinct innervation patterns of the mushroom bodies (Lee et al., 2020; Li et al., 2020). In analogy to the mushroom body phenotype, we assume that the CREa1 and CREa2 NBs prematurely cease their proliferation in *mbt^P1^* animals, resulting in a predominant failure to generate late-born PAM neurons. Under the condition that suitable markers for the CREa1/CREa2 NBs are available, the influence of Mbt on their proliferation can be verified. Also, the predominant loss of the larval pPAM1 neuron might relate to the birth order of the four pPAM neurons, but the identity of the progenitor cell is unknown so far. In conclusion, Mbt has a significant impact on the generation of PAM cells during development and is important for proper PAM cell function during adulthood, but has no neuroprotective role at old age.

This raises the question about the putative molecular functions of Mbt in PAM neurons to explain the age-dependent effects on climbing ability (Fig. 1A, Figs 3 and 4). In general, PAK proteins regulate a variety of cellular processes, including cytoskeleton remodeling, cell motility, mitosis, cell survival, gene transcription, steroid-receptor signaling, and neuronal development and plasticity (Civiero and Greggio, 2018; Kumar et al., 2017). Of special interest is the involvement of Mbt and PAK4 in Cadherin-mediated cell–cell adhesion (Faure et al., 2005; Pütz, 2019; Schneeberger and Raabe, 2003; Selamat et al., 2015; Wallace et al., 2010; Walther et al., 2016). Together with Canoe (vertebrate AF6 or AFDN), Rap1 and Bazooka (Par3), Mbt is one of the key players remodeling the zonula adherens (Walther et al., 2018, 2016). It is conceivable that this network also influences synaptic contacts, as rat AF6 localizes at synapses (Xie et al., 2008) and Rap1 is involved in the development and morphogenesis of the *Drosophila* neuromuscular junction (Ou et al., 2019). Furthermore, ectopic expression of human AF6 protects against DA dysfunction in *Drosophila* models of PD (Basil et al., 2017). Examining PD patients with regard to the pathways over-represented in genome-wide association studies or gene expression studies, adherens junction components appear among the top candidates (Edwards et al., 2011).

Looking at the neural circuit level, reactive locomotion control also involves other brain regions beside PAM neurons, e.g. the mushroom bodies (Sun et al., 2018). NP6510 PAM neurons mainly innervate specific regions of the mushroom body (Tanaka et al., 2008). These cell–cell contacts provide a critical interface with regard to age-dependent climbing performance and PD, because synaptic contacts are progressively depleted upon ectopic expression of a mutant form of α-synuclein in flies and, to a lesser degree, also in wild-type animals (Riemensperger et al., 2013). Interestingly, loss of synaptic contacts was not accompanied by PAM neuron cell death, a striking parallel to our findings with *mbt.* It will be an interesting starting point for further studies to examine age-dependent alterations of synaptic contacts between NP6510 PAM neurons and mushroom body neurons in *mbt* knockouts, with a particular focus on cell adhesion molecules. Because of the requirement of Mbt for generation of PAM and mushroom body neurons, such an experiment would rely on cell-type-specific knockout of *mbt* in the differentiated pre- and/or postsynaptic neurons.

In contrast to humans and rodents, in which PAK4 activity in DA neurons is reduced under PD disease conditions, which in turn induce DA cell loss (Danzer et al., 2007; Won et al., 2016), we were able to show, for the first time, that mutations in the *PAK4* homolog *mbt* trigger a variety of PD-related phenotypes in *Drosophila*. Mbt is required in DA PAM neurons for normal life expectancy and climbing performance. Mutations in *mbt* are not associated with progressive PAM neuron loss but most likely cause functional impairments. Given that also non-motor PD phenotypes such as sleep disturbances were observed, we consider *mbt*/*PAK4* as a new candidate gene for PD.

## MATERIALS AND METHODS

### Fly strains and genetics

Flies were kept at 25°C under a 12 h light, 12 h dark cycle unless otherwise noted. Canton S was used as a wild type. Because the *mbt^P1^* null mutation was originally induced in a *white* mutant background (Melzig et al., 1998), it was recombined in the Canton S background to minimize genetic variability. The genomic *mbt* transgene *P[gen-mbt]* (Pütz, 2019) was crossed into the cantonized *mbt^P1^* background to perform rescue experiments.

For targeted expression of *UAS-mbt* [encoding wild-type Mbt (Schneeberger and Raabe, 2003)] in DA neurons, the following Gal4 driver lines were used: *th-Gal4* [Gal4 under the control of the regulatory sequence of the *th* gene (Friggi-Grelin et al., 2003)], *Ddc-Gal4* [Gal4 driven by the promoter of Dopa decarboxylase (Li et al., 2000), Bloomington *Drosophila* Stock Center (BDSC), #7010], *R58E02-Gal4* [expressed in PAM neurons as well as glial cells of the optical lobes (Liu et al., 2012), received from Denis Pauls, University of Leipzig, Leipzig, Germany] or *NP6510-Gal4* [Gal4 expression in a subset of ∼15 DA PAM neurons (Riemensperger et al., 2013), Kyoto *Drosophila* Genetic Resource Center, #113956]. The following driver lines were used for control experiments: *DE-Gal4* (expresses Gal4 in the dorsal compartment of the eye imaginal disc, BDSC, #29650), *worniu-Gal4* (expresses Gal4 in neuroblasts, BDSC, #56553) and *ey^OK107^-Gal4* (drives expression among others in neuroblasts, mushroom bodies neurons and eye imaginal discs, BDSC, #854). To visualize Gal4 expression in DA PAM cell subgroups, we used the fluorescent FRET construct *UAS-Cameleon2.1* (Diegelmann et al., 2002; received from Thomas Riemensperger, University of Cologne, Cologne, Germany).

For cell-type-specific knockout of *mbt* by CRISPR/Cas9, we used the Heidelberg CRISPR Fly Design Library [HD_CFD (Port et al., 2020)] stocks *HD_CFD00807* [obtained from Vienna *Drosophila* Resource Center (VDRC), #341701], which expresses two different short guide (sg)RNAs targeting *mbt* under UAS control (referred to as *UAS-sgRNAmbt*) and *UAS-uM-Cas9* (referred to as *UAS-Cas9*), which expresses medium Cas9 levels by insertion of an upstream open-reading frame (HD_CFDtools003, VDRC, #340002 and HD_CFDtools008, VDRC, #340007).

### Climbing assay

To examine motor skills, the startle-induced negative geotaxis assay (climbing assay) was applied as previously described (Feany and Bender, 2000; Riemensperger et al., 2013). Throughout the experiment, flies were recorded by video. Cohorts of six to 20 flies were placed in 3.3 cm diameter and 16 cm high column-shaped vials. Per genotype, eight to 12 independent cohorts were analyzed. After tapping flies down, they respond reflexively by climbing. It is assumed that this reflex lasts for ∼10 s (Barone and Bohmann, 2013). Ten seconds after tapping, the climbing height of each fly was measured and the percentage of flies that had reached at least 1 cm or 8 cm was determined. Per cohort, the medium value of seven trials was taken for statistical analysis.

### Survival analysis and lifespan

To monitor life expectancy, cohorts of 70-200 1-day-old (0-24 h) male flies from each genotype were collected and maintained on regular food with a maximum of 20 flies per vial. Flies were transferred to fresh food at the latest every 10 days. Survivors were scored daily, and the survival functions including the 95% confidence interval were analyzed and plotted with the software OriginPro 2018 (OriginLab Corporation) using Kaplan–Meier survival curves.

### Locomotor activity and sleep analysis

Sleep in *Drosophila* is defined as a minimum of 5-10 min of inactivity in a locomotor assay (Gajula Balija et al., 2011; Gmeiner et al., 2013; Sun et al., 2016; Valadas et al., 2018). In this work, any inactivity phase of at least 5 min was considered as sleep. For analyses of daily activity and sleep patterns, the locomotor activity of male flies was monitored using the DAM (Trikinetics) system in a 25°C climate chamber. The mean activity profile, defined as the mean activity of examined flies within 1 min, was plotted over the course of the day for each genotype.

The following activity and sleep parameters were calculated for each single fly: total activity (light beam crosses within 24 h), diurnal/nocturnal index (calculation as in Julienne et al., 2017) and morning anticipation (calculation as in Valadas et al., 2018). In addition, total sleep (sum of sleep minutes within a given period) and sleep bout duration (mean duration of sleep phases within a given period) were calculated using a macro written in Microsoft Excel (Gmeiner et al., 2013). The activity bout duration (mean duration of awake phases within a given period) was calculated equivalent to sleep bout duration.

### Immunohistochemistry

Adult flies were fixed with 4% paraformaldehyde in PBS/0.15% Triton X-100 for 2.5 h and brains were afterwards dissected. Larval brains were dissected first and then fixed in 4% paraformaldehyde in PBS for 30 min. Brains were washed twice with PBT (0.3% Triton in PBS) before blocking in 5% normal goat serum (NGS) (5% NGS in PBT) at room temperature for 1 h, followed by incubation with primary antibodies at 4°C overnight. Primary antibodies were diluted in 5% NGS: mouse anti-TH clone LNC1 (1:200, Merck, MAB318), guinea pig anti-Lamin-DmO (1:300, kind gift from Georg Krohne, University of Würzburg, Würzburg, Germany), mouse anti-Fas2 (1:10, Developmental Studies Hybridoma Bank) or rabbit anti-GFP (1:1000, MoBiTec, A6455). Following three washing steps in PBT, brains were incubated for 4 h at room temperature with secondary antibodies: donkey anti-guinea-pig-DL650 (1:100, Thermo Fisher Scientific, SA5-10097) or donkey anti-guinea-pig-Cy3 (1:100, Dianova, 706-166-148); goat anti-rabbit-Alexa488 (1:100, Molecular Probes, A-11034); goat anti-mouse-Alexa488 (1:150, Dianova, 115-545-166), donkey anti-mouse-Cy3 (1:100, Dianova, 715-165-151) or donkey anti-mouse-Cy5 (1:100, Dianova, 715-175-151). After washing in PBT, brains were embedded in VectaShield (Vector Laboratories) and confocal images were recorded with a Leica TCS SPE microscope.

### FST and OFT

The FST was executed similarly to that described previously (Neckameyer and Nieto-Romero, 2015). For the learning phase, male flies were placed for 5 min in a round plastic well (diameter 3.2 cm, depth 1 cm) filled with 3 ml water/0.0025% Triton X-100 solution. Afterwards, the male fly was dried on a piece of tissue and placed back in a vial overnight together with a female at 25°C. 24 h later, the male fly was put back on the water/0.0025% Triton X-100 solution and filmed for 5 min. Survivors of this procedure who were able to walk in a dry vial afterwards were included in the analysis. For further data analysis, the immobile time was counted from the video.

Details on the OFT and other methods used to collect the data in Figs S1-S6 and Table S1 are provided in the Supplementary Materials and Methods.

### Ethics

All *Drosophila* experiments were performed according to the animal protection guidelines of the government of Unterfranken, State of Bavaria.

### Statistics

All statistical analyses were performed with the software OriginPro 2018 (OriginLab Corporation). For survival analysis, the Kaplan–Meier estimator and the log-rank test were used. All other data were analyzed with the Mann–Whitney test followed by Bonferroni correction for multiple comparisons. *P*<0.01 was considered significant.

## Supplementary Material

Supplementary information
