# Supplementary material for: Loss of p21-activated kinase Mbt/PAK4 causes Parkinson-like phenotypes in Drosophila
Source: Dis Model Mech. 2021 Jun 14;14(6):dmm047811. doi: 10.1242/dmm.047811 (PMC8246267; doi:10.1242/dmm.047811)
Supplement: Supplementary information [file dmm-14-047811-s1.pdf]

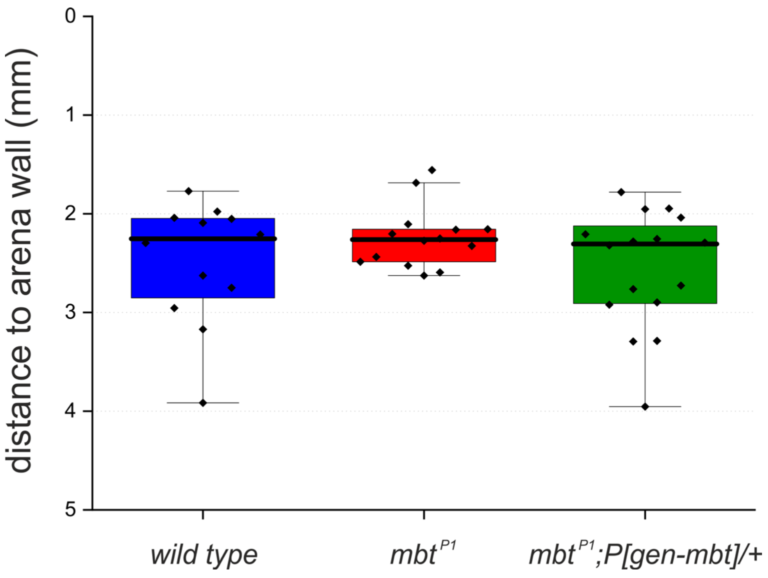

Fig. S1. Wall following behavior in the open field arena. Depicted is the distance to the arena wall of *wild type* (blue, n=12), *mbt^{P1}* (red, n=14) and *mbt^{P1};P[gen-mbt]/+* (green, n=16) animals. Each data point represents the mean distance to the arena wall of a single fly, determined from a 10-minute video. Although no significant differences between genotypes were observed, it is noticeable that the spread of the data is less for *mbt^{P1}* flies.

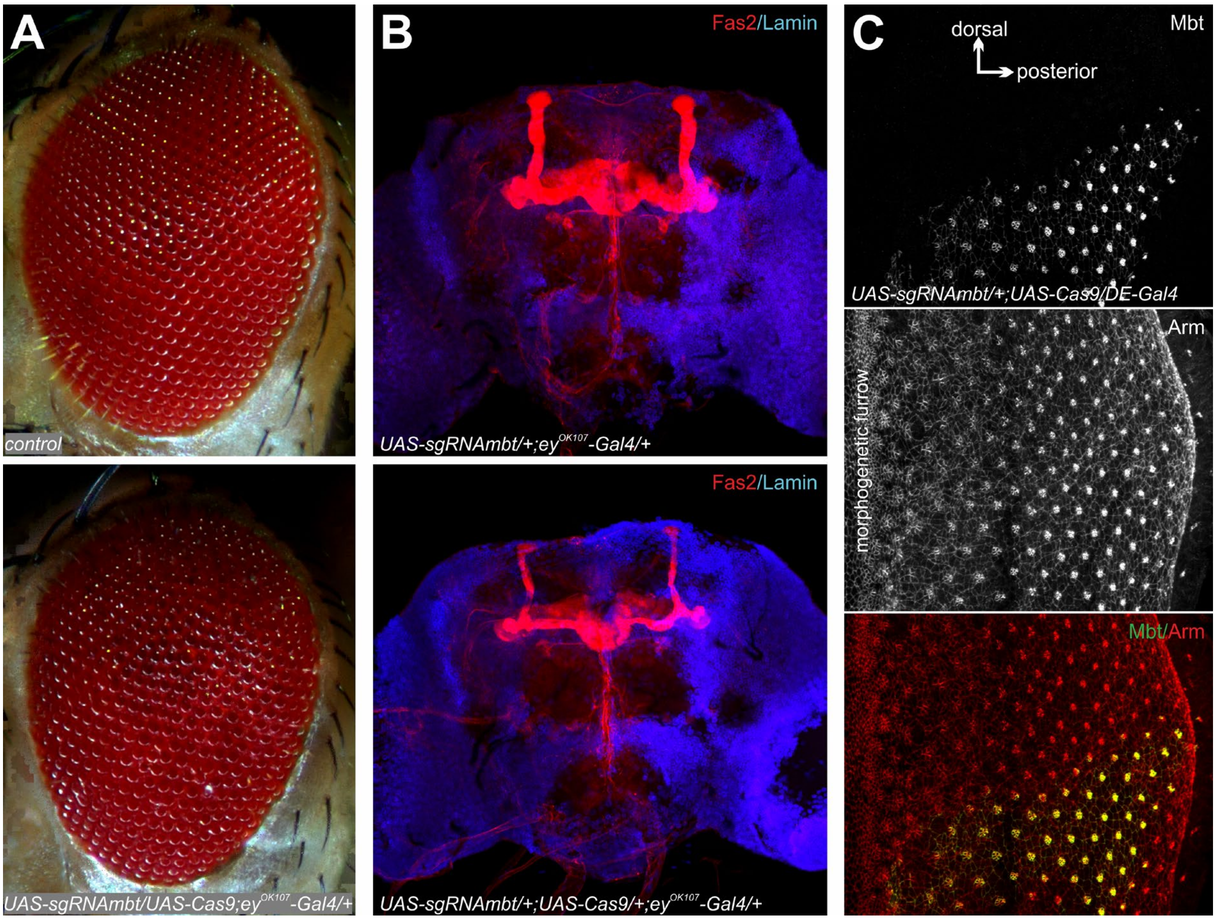

Fig. S2. Induction of known *mbt* mutant phenotypes by cell-type-specific *mbt* knockout using CRISPR/Cas9. Knockout of *mbt* in larval eye disc and mushroom body cell lineage with the ey^{OK107}*-Gal4* driver induces **A)** rough eyes and **B)** tiny mushroom bodies. Brains were stained with Fas2 (red) to label the mushroom body lobe system and Lamin (blue) as a nuclear membrane marker. **C)** *mbt* knockout using the *DE-Gal4* driver expressed in the dorsal half of the larval eye disc results in a corresponding loss of Mbt staining (green). Co-staining was with Armadillo (Arm, red), a marker for adherence junctions.

Disease Models & Mechanisms • Supplementary information

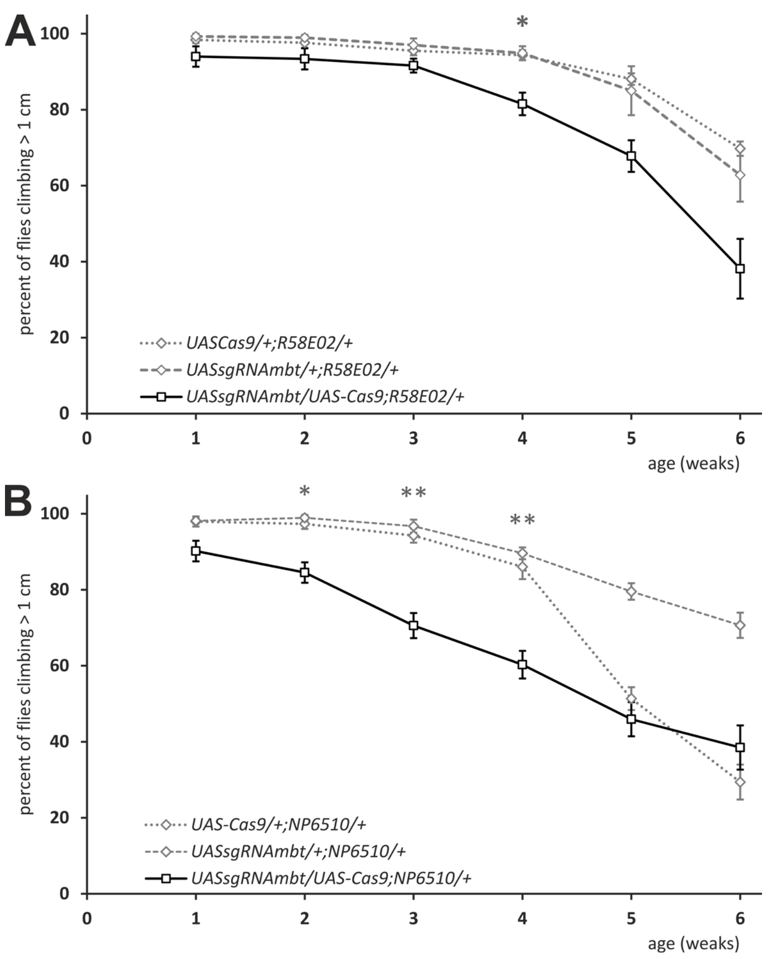

Fig. S3. Ability of flies with an *mbt* knockout in subsets of PAM neurons to initiate climbing. Depicted is the ability to climb 1 cm or more within 10 seconds over a period of six weeks. In **A)** flies of the genotype *UAS-sgRNAmbt/UAS-Cas9;R58E02-Gal4/+* are compared to controls *UAS-sgRNAmbt/+;R58E02-Gal4/+ and UAS-Cas9/+;R58E02-Gal4/+.* In **B)** *UAS-sgRNAmbt/UAS-Cas9;NP6510-Gal4/+* are compared to *UAS-sgRNAmbt/+;NP6510-Gal4/+* and *UAS-Cas9/+;NP6510-Gal4/+*. Shown are mean and SE of 7-12 independent cohorts, with the exception of the genotype *UAS-Cas9/+;R58E02-Gal4/+* (week 6 with only 5 cohorts). The P value is at least as depicted for each time point. *p<0,01, **p<0,001, ***p<0,0001 with the Mann-Whitney-Test followed by Bonferroni-correction.

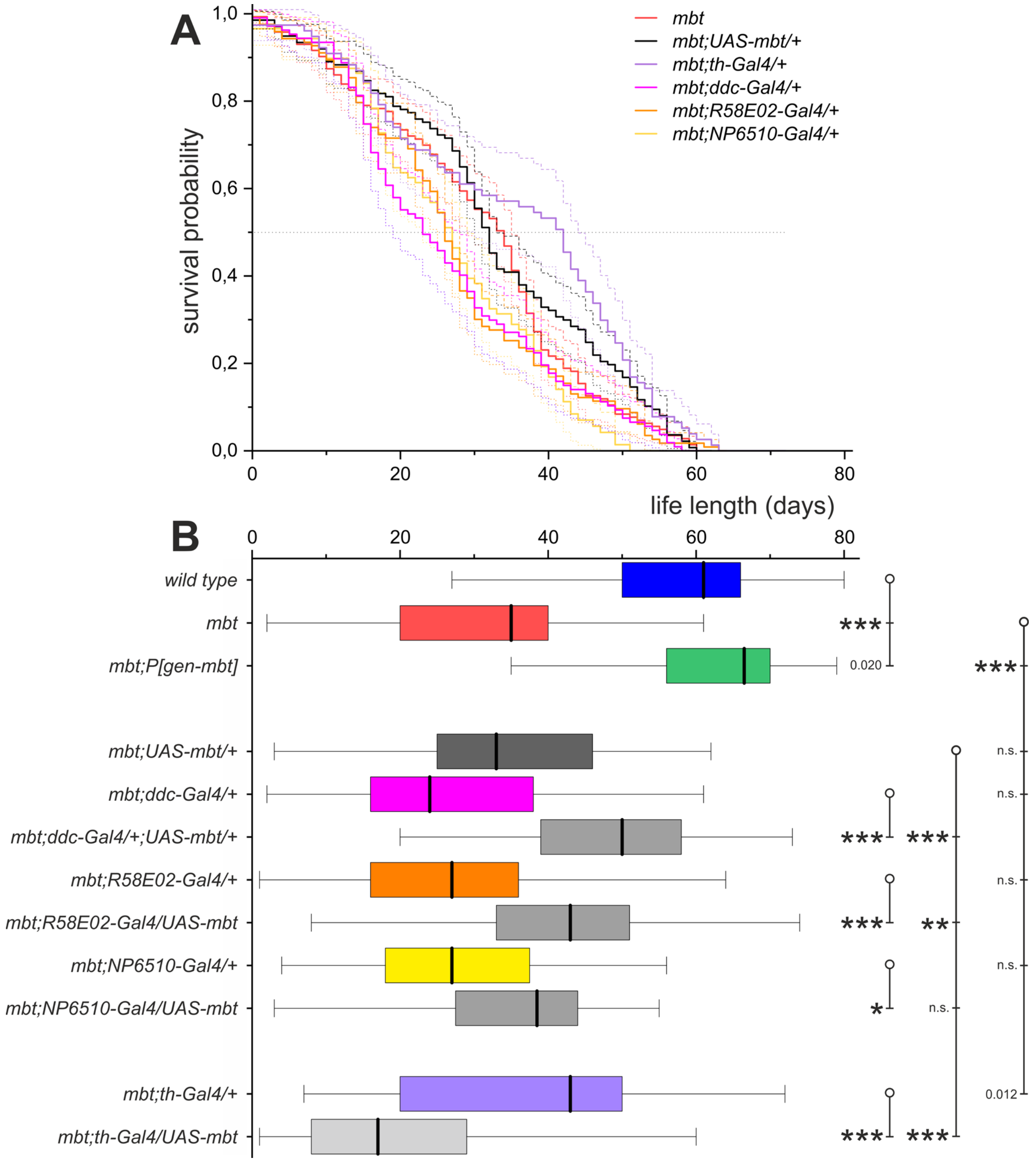

Fig. S4. **Statistical analysis of life span data. A)** depicts survival probability as curves including the 95% confidence intervals (dashed lines) of the controls belonging to Fig.5A-C the genotypes are *mbt*^{P1} (red, n=195), *mbt*^{P1}*;UAS-mbt/+* (black, n=137), *mbt*^{P1}*;NP6510-Gal4/+* (yellow, n=88), *mbt*^{P1}*;R58E02-Gal4/+* (orange, n=123), *mbt*^{P1}*;ddc-Gal4/+* (pink, n=170) and *mbt*^{P1}*;th-Gal4/+* (violet, n=77). None of the p-values calculated from the mbt^{P1};*Gal4*-controls compared to *mbt*^{P1} fell below the threshold of p<0.01 (see lower graph). **B)** shows life length depicted as box plots from the same genotypes as in the upper graph. For better statistical comparison, additionally the data from the genotypes *mbt*^{P1}*;NP6510-Gal4/UAS-mbt* (n=82), *mbt*^{P1}*;R58E02-Gal4/UAS-mbt* (n=127), *mbt*^{P1}*;ddc-Gal4/+;UAS-mbt/+* (n=72) and *mbt*^{P1}*;th-Gal4/UAS-mbt* (light gray, n=99) as well as wild-type (blue, n=195) and *mbt*^{P1}*;P[gen-mbt]* (green, n=102) are shown. Statistical analysis of the survival data was performed using Kaplan-Meier analysis and the log-rank test followed by Bonferroni-correction. Significant differences are characterized by *p<0,01, **p<0,001 and ***p<0,0001.

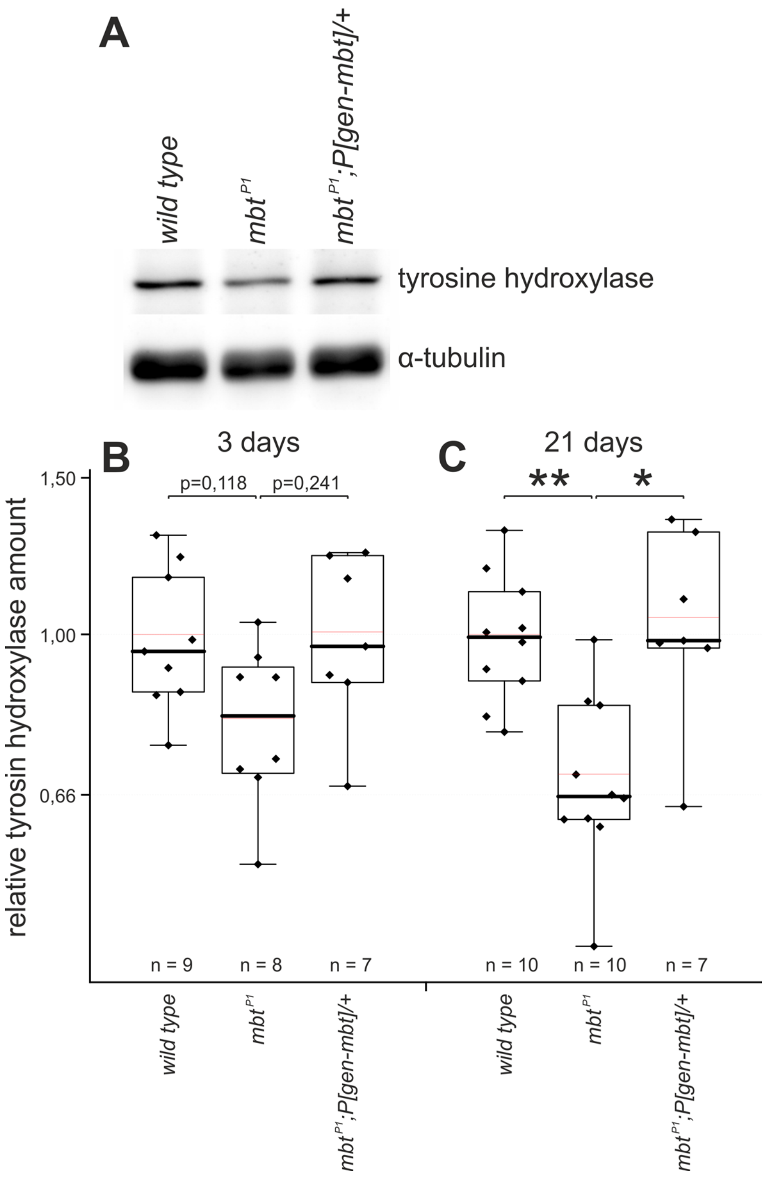

Fig. S5. Determination of tyrosine hydroxylase level in *mbt*^{P1} fly heads. A) Representative tyrosine hydroxylase (TH) western blot with its α-tubulin loading-control from heads of 3 day old flies of the indicated genotypes. **B-C)** Relative TH amount of fly heads form 3 days old (**B**) and 21 days old (**C**) animals, respectively. Each data point represents the TH amount of one sample normalized to its corresponding α-tubulin band and to the mean of wild-type samples running on the same gel. Statistical analysis was performed with the Mann-Whitney-Test followed by Bonferroni correction, significant differences are characterized by *p<0,05, **p<0,01.

```
dCRTC-PB        ---MANPRKFSEKIALQKQKQAEGTAEFERIMKEVYATKRDEPPANQKILDGLVGGQEVS      57
dCRTS-PA        ---MANPRKFSEKIALQKQKQAEGTAEFERIMKEVYATKRDEPPANQKILDGLVGGQEVS      57
hCRTC1          MATSNNPRKFSEKIALHNQKQAEETAAFEEVMKDLSLTRAARLQLQKSQY---------L      51
                     ***********::***** ** **.:**::  *:  .   ::.

dCRTC-PB        QSSPGAGNGTGGGGSGSGSGASGGGAS---------PDGLG------GGGGSPTAYRESR      102
dCRTS-PA        QSSPGAGNGTGGGGSGSGSGASGGGAS---------PDGLG------GGGGSPTAYRESR      102
hCRTC1          QLGPSRGQYYGG--SLPNVNQIGSGTMDLPFQTPFQSSGLDTSRTTRHHGLVDRVYRE-R      108
                * .*. *:  **  *  . .  *.*:           .**.        *    .*** *

dCRTC-PB        GRSVGVGPMRRPS-----ERKQDRSPYGSSSTQQTLDNGQLNPHLLGPPTAESLWRRSSS      157
dCRTS-PA        GRSVGVGPMRRPS-----ERKQDRSPYGSSSTQQTLDNGQLNPHLLGPPTAESLWRRSSS      157
hCRTC1          GR--LGSPHRRPLSVDKHGRQADSCPYGT---------------MYLSPPADTSWRRTNS      151
                **    .* ***       *: * .***:               :   * *:: ***:.*

dCRTC-PB        DSALHQSALVAGFNSDVNSMGANYQQQQHQQQQQPGQPRSHSPHHGINRTMSPQAQRRKS      217
dCRTS-PA        DSALHQSALVAGFNSDVNSMGANYQQQQHQQQQQPGQPRSHSPHHGINRTMSPQAQRRKS      217
hCRTC1          DSALHQSTMTPTQPESFSSGSQDVHQKRVLLLTVPGMEETTS---EADKNLSKQAWDTKK      208
                *******::.    ....* . : :*::      **  .: *     ::.:* **   *.

dCRTC-PB        PLLQPHQLQLQQLQQQQQQMQHQHQLHQQLQMQQLQQHQQQHQQQQQQQNTPYNNAKFTN      277
dCRTS-PA        PLLQPHQLQLQQLQQQQQQMQHQHQLHQQLQMQQLQQHQQQHQQQQQQQNTPYNNAKFTN      277
hCRTC1          TGSRPKSCEVPGIN--------------------IF----------------PSADQENTT      233
                   :*:. ::  ::                    :                *  : : *.

dCRTC-PB        PVFRPLQDQVNFANTGSLPDLTALQNYGPQQQQQQSQQQPSQQQQQLQQTLSPVMSPHNH      337
dCRTS-PA        PVFRPLQDQVNFANTGSLPDLTALQNYGPQQQQQQSQQQPSQQQQQLQQTLSPVMSPHNH      337
hCRTC1          A-L----IPATHNTGGSLPDLTNIHFPSPLPTPLDPE-EP----------TFPALSSSSS      277
                  :      ... . ******* ::  .*     : : :*            *.:*  .

dCRTC-PB        RRERDQSPSPFS-PAGGGGGAGPGSPYQQQQHSPTGNTQQQ-----QQQHQQPSNSPHLS      391
dCRTS-PA        RRERDQSPSPFS-PAGGGGGAGPGSPYQQQQHSPTGNTQQQ-----QQQHQQPSNSPHLS      391
hCRTC1          T---GNLAANLTHLGIGGAGQGMSTPGSSPQHRPAGVSPLSLSTEARRQQASPTLSPLSP      334
                    .:  : ::  . **.* * .:* .. ** *:* :  .     ::*: .*: **
```

Fig. S6. Sequence comparison of the N-terminal part of human CRTC1 with the two *Drosophila melanogaster* CRTC isoforms. Serine 215 (blue) is not conserved in *Drosophila melanogaster* CRTC.

Table S1. Phenotypes induced by cell-type-specific *mbt* knockout with CRISPR/Cas9 using different *Gal4* driver lines.

Gal4 driver line	expression	tiny mushroom bodies	rough eyes	less PAM cells	climbing impairments
ey^OK107-Gal4	e.g. eye disc cells, mushroom body	yes	yes		
DE-Gal4	dorsal eye disc		yes		
R58E02-Gal4	about 80% of the PAM neurons	no	no	no	yes
NP6510-Gal4	PAM subgroup		no		yes
wor-Gal4	neuroblasts	yes	no	yes	

Supplementary Materials and Methods

Open field test - The open field test to determine the wall following behavior was carried out similar as described previously (Chen et al., 2014; Mohammad et al., 2016). In detail, three day old flies were immobilized on ice for 5 minutes and then placed individually in open field arenas (diameter 1 cm, height 1.7 mm). A 10-minute video was then recorded at 5 frames per second. The path of the flies was tracked using Caltech Multiple Walking Fly Tracker (CRTAX (Branson et al., 2009)). The coordinates at which the fly is located in the respective frame were exported as a csv file. From the coordinates of the fly, the distance to the center of the arena or the arena wall was determined at any time point using an in-house MatLab script.

Protein extracts, PAGE and Western blot - For each sample, five adult heads were dissected, the head capsules were mechanically broken and lysed directly in 20µL doubly concentrated SDS-PAGE sample buffer by boiling for 5 min at 90°C. Separation on 10% SDS-polyacrylamide gels was followed by transfer of the proteins to nitrocellulose by tank-blotting. Membranes were blocked with 5% dry milk in TBST (0.1% Tween20 in TBS). Primary antibodies were diluted in blocking solution: mouse anti-TH (1:2000, Merck, MAB318), mouse anti-α-Tubulin (1:5000, Sigma, T9026). Protein bands were detected with a ChemoCam (Intas) using HRP-coupled secondary antibodies (GE Healthcare) and ECL Prime Western Blotting Detection Reagent (GE Healthcare).

Sequence comparison - Multiple protein sequences alignments were accomplished using Clustal Omega (Sievers et al., 2011).

Immunostainings of larval eye imaginal discs - Eye discs were dissected from 3rd instar larvae and fixed in PLP (75 mM lysin, 10 mM $NaIO_4$, 2.8% paraformaldehyde in 30 mM sodiumphosphate buffer pH 6.8) for 20 minutes on ice. The further steps correspond to the brain staining. Rabbit anti-Mbt (Schneeberger and Raabe, 2003) and mouse anti-Armadillo (1:33, Developmental Studies Hybridoma Bank, N27A1) were used as primary antibodies, followed by washing steps and incubation with the secondary antibodies donkey anti-mouse-Cy5 (1:100, Dianova, 715-175-151) and goat anti-rabbit-Alexa488 (1:100, Molecular Probes, A-11034).

Photographic recording of eyes - Flies were collected in clean vials and briefly frozen at -20°C. Eyes were photographed with a Zeiss Discovery V8 stereomicroscope fitted with a 1.5x lens and processed with Axiovision Extended Focus software.

Supplementary References

Branson, K., Robie, A. A., Bender, J., Perona, P. and Dickinson, M. H. (2009). High-throughput ethomics in large groups of Drosophila. *Nat Methods* **6**, 451-7.

Chen, A. Y., Wilburn, P., Hao, X. and Tully, T. (2014). Walking deficits and centrophobism in an alpha-synuclein fly model of Parkinson's disease. *Genes Brain Behav* **13**, 812-820.

Mohammad, F., Aryal, S., Ho, J., Stewart, J. C., Norman, N. A., Tan, T. L., Eisaka, A. and Claridge-Chang, A. (2016). Ancient Anxiety Pathways Influence Drosophila Defense Behaviors. *Curr Biol* **26**, 981-986.

Schneeberger, D. and Raabe, T. (2003). Mbt, a Drosophila PAK protein, combines with Cdc42 to regulate photoreceptor cell morphogenesis. *Development* **130**, 427-437.

Sievers, F., Wilm, A., Dineen, D., Gibson, T. J., Karplus, K., Li, W., Lopez, R., McWilliam, H., Remmert, M., Soding, J. et al. (2011). Fast, scalable generation of high-quality protein multiple sequence alignments using Clustal Omega. *Mol Syst Biol* **7**, 539.
